# Organic electro-scattering antenna: Wireless and multisite probing of electrical potentials with high spatial resolution

**DOI:** 10.1126/sciadv.adr8380

**Published:** 2024-12-20

**Authors:** Benoit Desbiolles, Jad Hanna, Raphael Ausilio, Marta Airaghi Leccardi, Yang Yu, Deblina Sarkar

**Affiliations:** ^1^ Nano-Cybernetic Biotrek, Media Lab, Massachusetts Institute of Technology, Cambridge, MA 02139, USA.; ^2^ Raith, Troy, NY 12180, USA.

## Abstract

Monitoring electrical potentials with high recording site density and micrometer spatial resolution in liquid is critical in biosensing. Organic electronic materials have driven remarkable advances in the field because of their unique material properties, yet limitations in spatial resolution and recording density remain. Here, we introduce organic electro-scattering antennas (OCEANs) for wireless, light-based probing of electrical signals with micrometer spatial resolution, potentially from thousands of sites. The technology relies on the unique dependence of poly(3,4-ethylenedioxythiophene):polystyrene sulfonate light scattering properties to its doping level. Electro-optic characteristics of individual antennas varying in diameters and operating voltages were systematically characterized in saline solution. Signal-to-noise ratios up to 48 were achieved in response to 100-mV stimuli, with 2.5-mV detection limits. OCEANs demonstrated millisecond time constants and exceptional long-term stability, enabling continuous recordings over 10 hours. By offering spatial resolution of 5 μm and a recording density of 4 × 10^6^ cm^−2^, OCEANs unlock new readout capabilities, potentially accelerating fundamental and clinical research.

## Teaser

Organic antennas enable wireless detection of electrical potentials with micrometer resolution for biosensing applications.

## INTRODUCTION

The ability to probe electrical potentials with high recording site density and micrometer spatial resolution in a wet environment is a holy grail of biosensing. It can unlock unparalleled opportunities for enhanced multiplexing, throughput, and spatial resolution across a wide range of applications, including biomolecule detection (*1*, *2*), DNA sequencing (*3*), impedance sensing (*4*), functional imaging (*5*), and electrophysiology (*6*–*8*). Over recent decades, organic electronic materials (OEMs) have led to remarkable advances toward this goal (*9*). Their unique electrochemical properties in aqueous electrolytes make them materials of choice for developing highly sensitive reporters of bioelectrical signals.

For instance, OEMs’ mixed ionic and electronic conductivities enabled neuroprosthetics with enhanced tissue-electrode interfaces, facilitating electrical recording and stimulation (*10*–*12*). In addition, electrical modulation of OEM doping levels led to the development of organic electrochemical transistors (OECTs) (*13*), a class of highly sensitive biosensors extensively used in various applications, particularly electrophysiology (*14*–*16*), impedance sensing (*17*–*21*), and analyte detection (*22*–*24*). The dependence of OEM optical absorbance properties on doping levels—and, therefore, on voltage—permitted wireless monitoring of cellular electrophysiological activities (*25*, *26*). On the basis of this principle, the electrochromic optical recording device (ECORE) enabled high-sensitivity single-point measurements with single-cell resolution. More generally, OEM doping levels affect not only their optical absorbance but also their complex refractive index (*27*). Recently, electrically switchable optical diffraction gratings were developed, leveraging this principle (*28*). When coated onto plasmonic nanoantennas, OEMs were also demonstrated to enable electrical modulation of the plasmonic resonance (*29*–*31*). Arrays of these electroplasmonic nanoantennas enabled wireless probing of cardiomyocyte activity from a single recording site of a few hundred micrometers in diameter. Beyond electroplasmonic antennas, electrochemically modulated plasmonic nanoantennas fully composed of OEMs were successfully developed. Notably, poly(3,4-ethylenedioxythiophene):polystyrene sulfonate (PEDOT:PSS) displays a metallic behavior at high doping levels with a negative real dielectric permittivity, enabling plasmonic resonance in the infrared domain (*32*). Upon dedoping, it exhibits the characteristics of a dielectric (i.e., positive real dielectric permittivity), quenching the plasmonic resonance of the antenna. Arrays of polymer plasmonic nanoantennas were recently implemented in the context of integrated photonics to modulate light beam intensities electrically (*33*–*35*).

While OEMs have led to remarkable advances in biosensing, the spatial resolution and recording site density of organic biosensors remain problematic. For OECTs, and more generally, for microelectrode-based sensors, the spatial resolution is typically limited to a few hundred micrometers and the number of recording sites to a few tens because of the conductive traces connecting each sensing unit to its electrical instrumentation. Nevertheless, studying complex biological systems requires enhanced spatial resolution to accurately recapitulate physiological processes occurring at subcellular scales. Specifically, single-micrometer resolution across thousands of recording sites is necessary to enable functional readouts with spatial context and open new avenues in biosensing. Yet, none of the existing technologies have enabled such features.

This work introduces an innovative organic electro-scattering antenna (OCEAN) to wirelessly probe small potential fluctuations using visible light in a physiological solution. OCEANs leverage the unique dependence of PEDOT:PSS light scattering on voltage to enable wireless sensing of millivolt electrical signals, potentially from thousands of recording sites and with micrometer resolution. These features make OCEANs unique compared to state-of-the-art technologies and position them as a central class of devices to perform multisite probing of bioelectrical signals with high spatial resolution, potentially opening new avenues in spatial biology.

First, we developed a theoretical model describing how external potentials applied across PEDOT:PSS OCEANs affect their doping level and complex permittivity in a physiological environment. We subsequently leveraged this model to study how changes in permittivity affect their scattering spectrum and dynamic signal in the visible domain. Furthermore, we established a robust nanofabrication process to manufacture OCEAN arrays of different dimensions and studied their electro-optic modulation characteristics in terms of sensitivity, noise, signal-to-noise ratio (SNR), limit of detection, time constant, and long-term stability. We demonstrate that single antennas can be used to monitor 100-mV voltage pulses wirelessly with SNRs up to 48 and a limit of detection approaching 2.5 mV at millisecond timescales and 5-μm spatial resolution. The advantages and limitations of OCEANs compared to state-of-the-art technologies are described in the Discussion.

## RESULTS

### The OCEAN concept

**Fig. 1.**
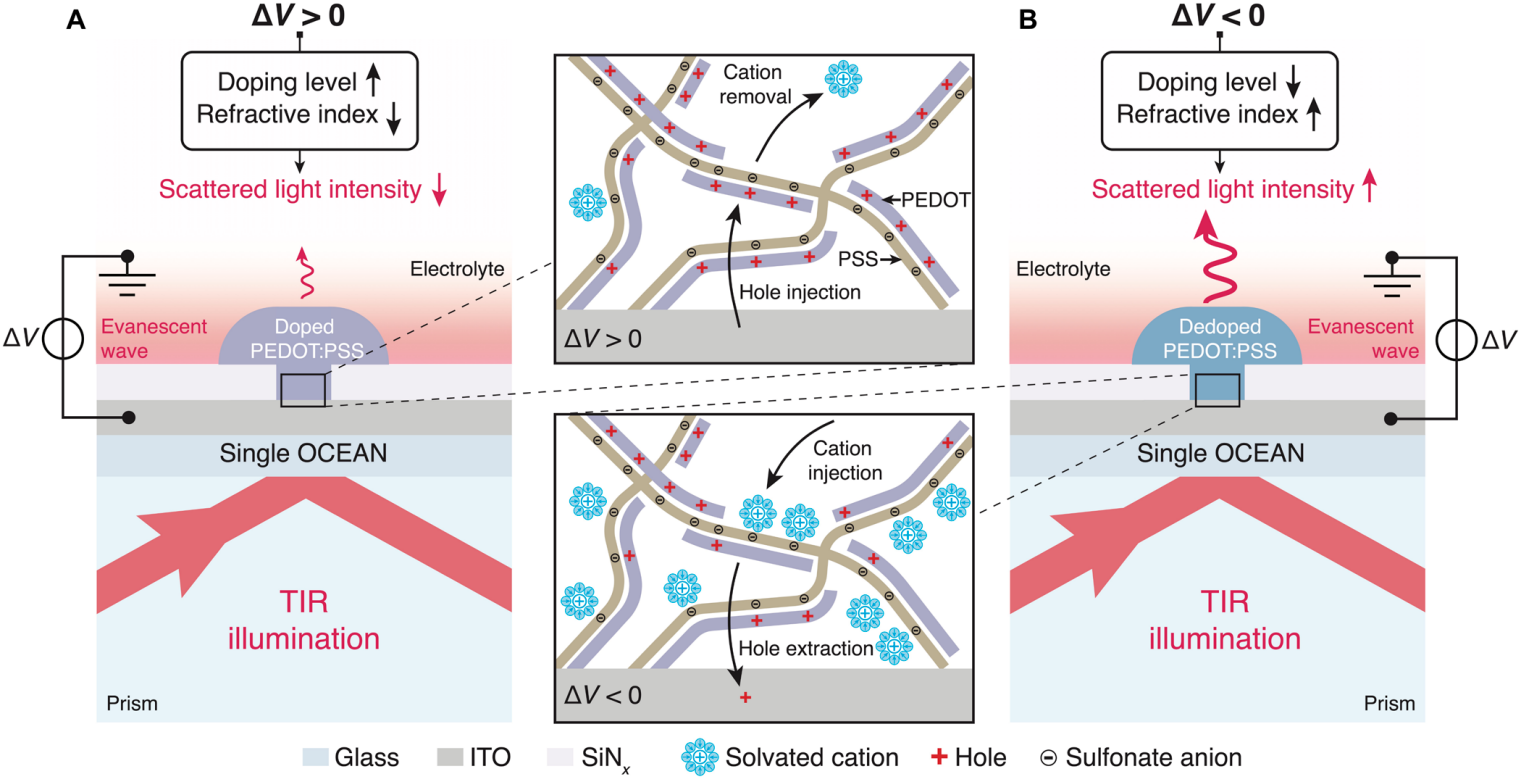
Concept of OCEAN. (**A**) Schematic cross-sectional representation of a single OCEAN under positive voltage bias. Under this condition, holes are injected from the ITO electrode into the PEDOT chains, increasing its doping level and releasing the solvated cations that were previously compensating for the fixed sulfonate ion charges in the PSS strands (inset, top). The augmented charge density in PEDOT decreases its refractive index, therefore reducing its scattering properties. (**B**) Under negative voltage bias, solvated cations are injected into PEDOT:PSS from the electrolyte and electrostatically compensate for the fixed sulfonate anion charges, subsequently enabling hole extraction through the ITO electrode (inset, bottom). In its dedoped state, PEDOT shows an augmented refractive index and, consequently, enhanced scattering properties. In each configuration, the light scattered by OCEANs originates from an evanescent wave generated by total internal reflection (TIR). Schematic representations in the insets were adapted with permission from Proctor *et al.* (*55*).

Figure 1 illustrates the concept of OCEAN. It is composed of a PEDOT:PSS mushroom-like structure supported by an optically transparent and electrically conductive indium tin oxide (ITO)–coated glass substrate patterned on an electrically insulating silicon nitride (SiN*_x_*) layer. The cap and stem diameters are typically 1 μm and 250 nm, respectively. Upon positive bias voltage in the ITO layer, holes are injected in the PEDOT strands, increasing its doping level and decreasing its refractive index in the visible domain (Fig. 1A). Conversely, a negative electrical stimulus across the OCEAN causes a reduction in its doping level and augments its refractive index within the same spectral window (Fig. 1B). Furthermore, the scattering of a dielectric particle is directly related to its refractive index and that of the surrounding medium. When the refractive index of the particle is larger than that of the medium, its scattering is larger when its refractive index is higher. As a result, the light scattered by OCEANs is anticipated to be brighter at negative bias voltages—when PEDOT:PSS is dedoped—and vice versa. Practically, isolating light scattered by OCEANs requires the geometric separation of illumination and detection path, which can be achieved using a variety of dark-field imaging modalities (*36*).

### Modeling of OCEANs

The scattering cross section of a particle—defined as the ratio between the scattered light power and the incident light intensity—is highly dependent on its complex refractive index—or complex permittivity—as well as that of the embedding medium (see section S1.1 for details about the relationship between complex refractive index and complex permittivity) (*37*). While analytical expressions relating permittivity to the scattering cross section can be derived in isolated settings [small-size approximation for Rayleigh scattering (*38*) and spherical particle geometry for Mie theory (*39*)], more complex geometries require numerical treatment. In the following section, we highlight our three-step modeling efforts. First, we study how external voltage biases modulate the complex permittivity of PEDOT:PSS via electrochemical doping and dedoping processes in phosphate-buffered saline (PBS) solution. Next, we conduct numerical simulations of our proposed OCEAN structure to investigate how changes in OCEAN permittivity relate to changes in scattering cross sections. Last, we use an equivalent circuit model to extract dynamic scattering responses to applied voltages, providing insights into OCEAN’s operation bandwidth.

At equilibrium, positive charge carriers in the PEDOT backbone (i.e., polarons and bipolarons) are stabilized via electrostatic interactions with negatively charged PSS strands. As a result, PEDOT:PSS is naturally in a highly conductive (i.e., doped) state. Under negative voltage, cations from the electrolyte infuse into PEDOT:PSS, neutralize the negatively charged PSS strands, and locally reduce PEDOT into its dedoped state, making it less conductive and changing its dielectric properties (see section S1.1). In its fully doped state, the complex permittivity of PEDOT:PSS can be described by a Drude model, which is consistent with a metallic behavior (fig. S2, A and B). On the other hand, when PEDOT:PSS is fully dedoped, its permittivity is best fitted by a combination of Lorentz and Tauc-Lorentz functions, consistent with a purely dielectric behavior. Note that these permittivities vary linearly with their respective volumetric density of charge carriers. Consequently, computing the complex permittivities for intermediate doping levels comes down to a linear interpolation between the fully doped and dedoped permittivities. Complex permittivities of PEDOT:PSS for different doping levels are presented and discussed in detail in section S1.2. The relationship between the PEDOT:PSS doping levels and the corresponding voltage amplitudes applied across it in PBS was experimentally established and is also reviewed in section S1.3.

**Fig. 2.**
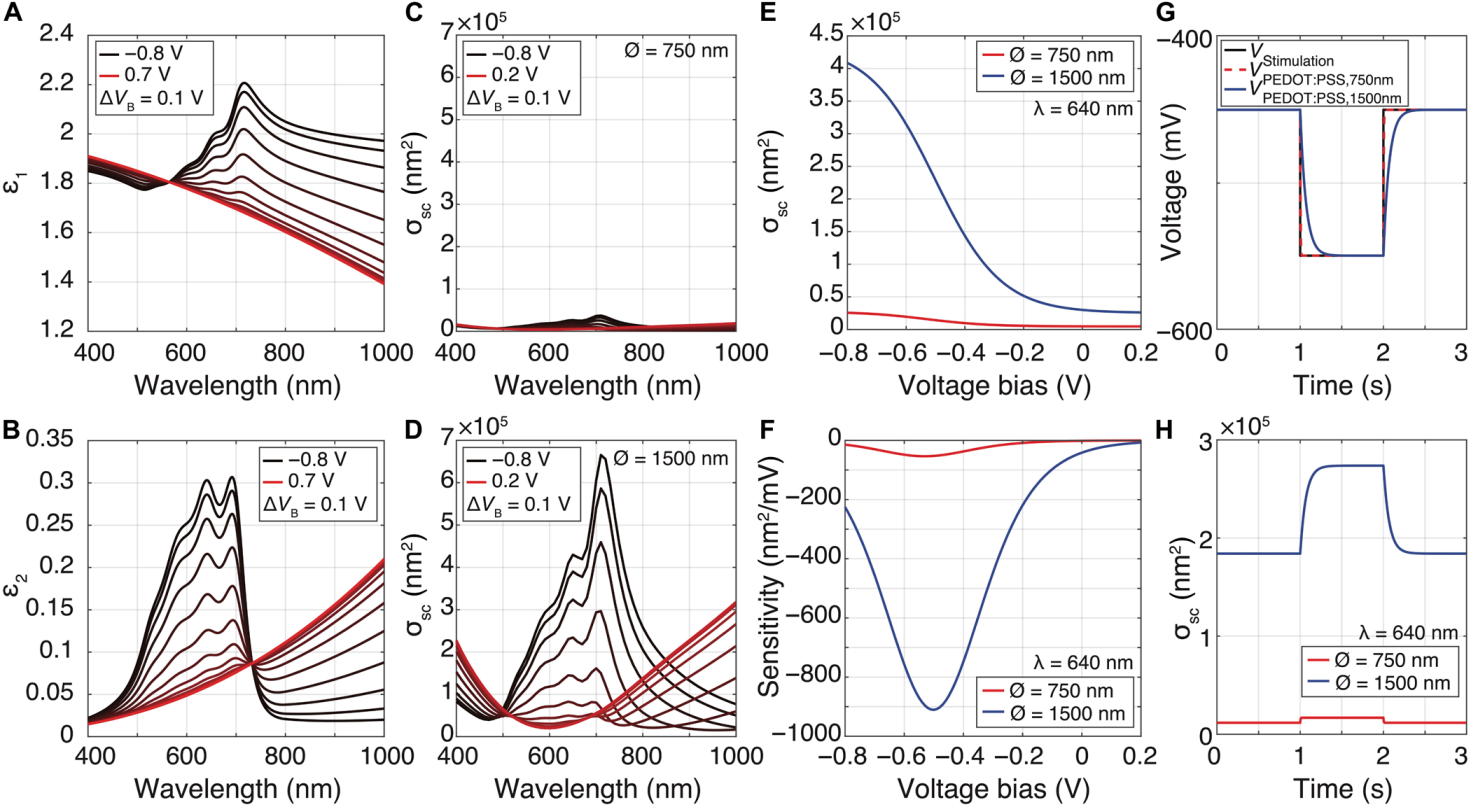
Modeling of single OCEAN electro-optic characteristics. (**A**) Real (ε_1_) and (**B**) imaginary (ε_2_) parts of PEDOT:PSS permittivity for different bias voltages *V*_B_ in PBS versus Ag/AgCl reference electrode. Simulated scattering cross-sectional (σ_sc_) spectra for a single PEDOT:PSS OCEAN with cap diameters of (**C**) 750 nm and (**D**) 1500 nm in response to voltage biases ranging from 0.2 to −0.8 V in PBS. (**E**) Scattering cross section or (**F**) sensitivity against voltage biases simulated for single OCEANs with cap diameters of 750 nm (red) and 1500 nm (blue) at a wavelength of 640 nm. (**G**) Dynamic voltages anticipated across 750- and 1500-nm-diameter OCEANs (*V*_PEDOT:PSS,750nm_ and *V*_PEDOT:PSS,1500nm_, respectively) in response to an applied −100-mV voltage pulse (*V*_Stimulation_) with respect to a −0.45-V bias voltage. (**H**) Dynamic electro-optic modulation simulated in response to the stimulation voltage pulse described in (G) for the two different OCEAN geometries tested.

Figure 2 (A and B) shows the PEDOT:PSS permittivity’s real and imaginary parts under different voltage bias conditions in PBS. The resulting scattering cross sections simulated for a single OCEAN with diameters of 750 and 1500 nm are presented in Fig. 2 (C and D, respectively). Predictably, larger OCEANs yielded larger scattering magnitudes. At negative voltages, the real part of PEDOT:PSS permittivity increased, especially for wavelengths near 700 nm, thus enhancing its scattering properties and leading to brighter OCEANs within this spectral window. Figure 2E highlights how static voltage biases applied across OCEANs influence their scattering cross section at a wavelength of 640 nm. Because the variation of PEDOT:PSS permittivity is not linear with voltage, the OCEAN sensitivity—defined here as the variation of scattering cross section with respect to voltage—is expected to be maximal at operating biases near −0.5 V (Fig. 2F).

Based on the electrical equivalent circuit of an OCEAN detailed in section S3, the dynamic voltage trace transmitted across the PEDOT:PSS structure in response to a −100-mV-amplitude voltage pulse applied between the ITO and the bath was computed at an optimal operating bias of −0.5 V (Fig. 2G). Because of their larger volumetric capacitance, larger OCEANs exhibit slower dynamics than their smaller counterparts (fig. S6). Figure 2H presents how these electrical stimuli were simulated to modulate the optical signal of single OCEANs. A negative voltage pulse leads to a positive variation of the optical signal, highlighting the central role of the real part of PEDOT:PSS permittivity in light scattering.

Our simulations demonstrate that the intensity of the light scattered by single OCEANs can be electrochemically modulated by applying voltage stimuli with millivolt amplitudes and millisecond kinetics.

### Nanofabrication of OCEANs

**Fig. 3.**
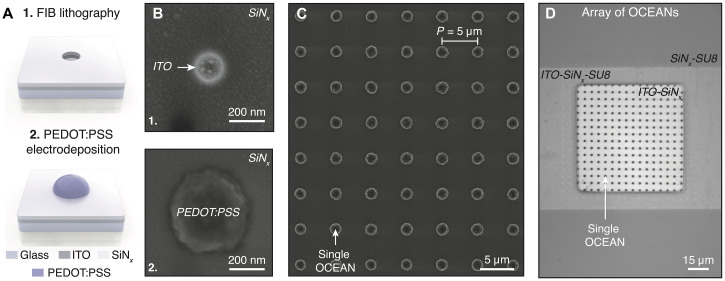
Nanofabrication of OCEANs. (**A**) Schematic representation highlighting the two main steps of nanofabrication process flow developed to manufacture OCEANs. (**B**) SEM images of nanostructures following FIB patterning (top) and PEDOT:PSS electrodeposition (bottom). (**C**) SEM micrograph of an array of OCEANs. (**D**) Bright-field microscopy image of an ITO-SiN*_x_* pad decorated with an array of OCEANs on an otherwise SiN*_x_*-SU8–passivated substrate.

Arrays of OCEANs were manufactured following the nanofabrication process flow presented in fig. S3. First, ITO traces and pads were patterned on an otherwise insulating glass substrate by photolithography and reactive ion etching (fig. S3, steps 1 and 2). To passivate the ITO structures, a 50-nm-thick SiN*_x_* layer was deposited by plasma-enhanced chemical vapor deposition and coated with a 3-μm-thick film of SU8. Square openings (80 μm) were patterned by photolithography in SU8 to access the ITO-SiN*_x_* pads and defined the location of the OCEAN arrays (fig. S3, steps 3 and 4). As highlighted in Fig. 3A, focused-ion beam (FIB) lithography was then performed to pattern arrays of nanoholes in the exposed SiN*_x_* layer (diameter ranging from 100 to 500 nm and pitch of 5 μm), locally revealing the ITO layer underneath. Last, protruding PEDOT:PSS structures were grown through each cavity by electrodeposition and formed arrays of OCEANs (fig. S3, steps 5 and 6). The influence of the electrodeposition time on the OCEAN diameter is described in section S2.2. Figure 3 (B to D) shows scanning electron microscopy (SEM) and bright-field microscopy images of the resulting structures at different steps of the fabrication process.

Each chip comprised 25 arrays, each composed of 16 by 16 OCEANs. Each individual array was electrically addressed independently, and light scattered by individual OCEANs within the arrays was collected at the single-unit level. Chips were mounted on custom-designed printed circuit boards (PCBs) and assembled with glass rings to facilitate electrical interfacing and experiments in liquid (see fig. S3B and section S2.1 for additional details).

### Electro-optic characterization of OCEANs

Electro-optic properties of OCEANs grown through 250-nm-diameter openings and for electrodeposition times (*t*_ED_) of 30, 45, and 60 s were investigated in this section. The corresponding cap diameters were 0.7, 1.4, and 1.8 μm, respectively (section S2.2).

First, relative irradiance spectra of OCEAN arrays were characterized in PBS for bias voltages ranging from 0 to −0.7 V using dark-field spectroscopy (section S4.1). As presented in fig. S7, larger negative biases lead to brighter OCEANs. The scattered light intensity peaked at wavelengths neighboring 700 nm. These experimental results confirm the influence of PEDOT doping and dedoping on the scattering properties of OCEANs and demonstrate our theoretical predictions.

**Fig. 4.**
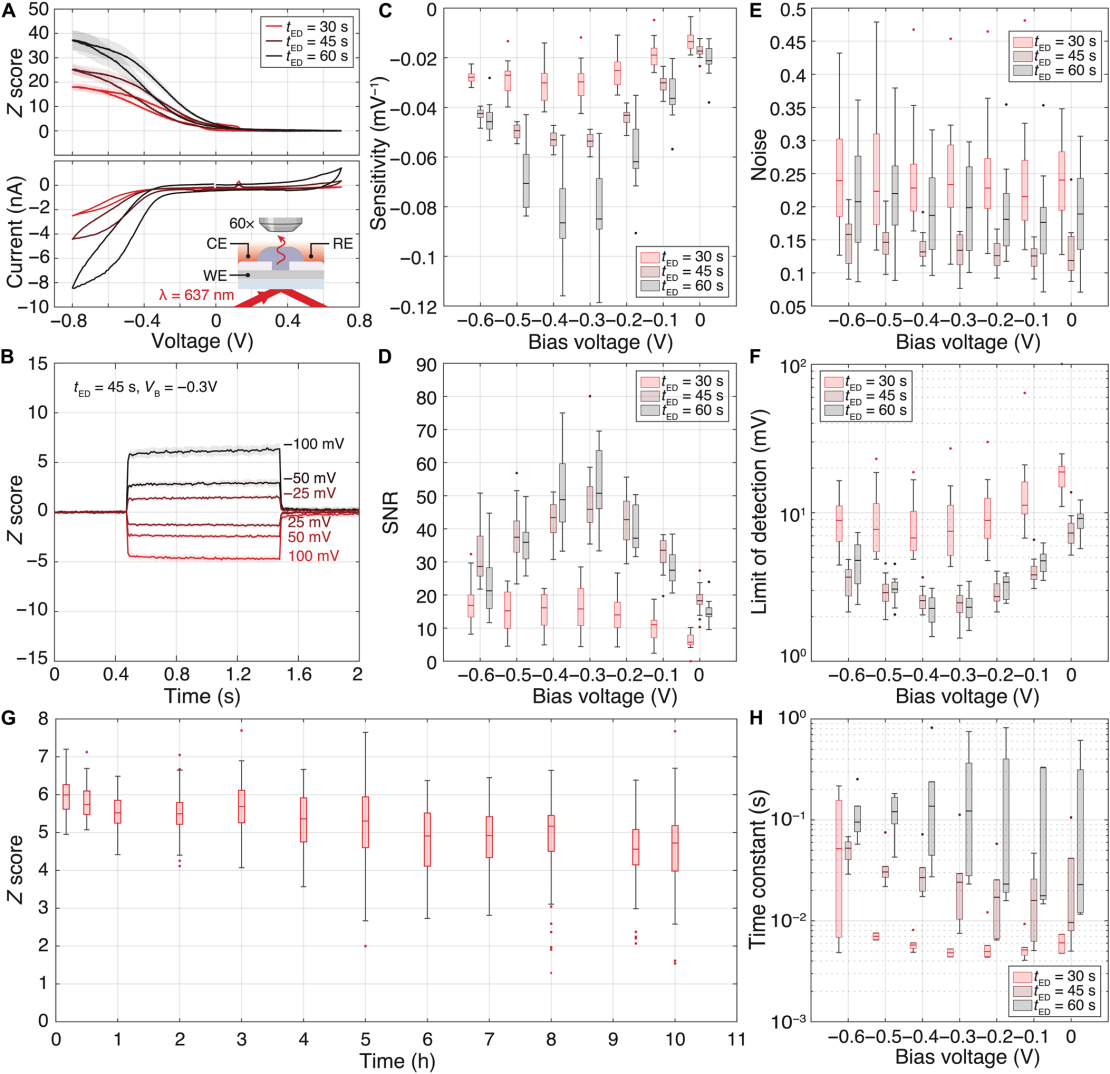
Electro-optic characterization of OCEANs. (**A**) Optical and electrical voltammograms. Relative variation of scattered light intensity (*Z* score) emitted by single OCEANs of different diameters in response to voltage sweeps (top, *N* = 16, means ± SD). Corresponding electrical current measured at the array level for each geometry tested (bottom). The inset shows a schematic representation of the experimental setup used for electro-optic characterization. (**B**) *Z* score modulation in response to voltage pulses of amplitudes between −100 and 100 mV measured for single OCEANs (*N* = 16, means ± SD). Box charts summarizing the (**C**) sensitivity, (**D**) SNR for 100-mV voltage pulses, (**E**) optical noise, and (**F**) limit of detection for single OCEANs of different geometries and operating at voltage biases ranging from 0 to −0.6 V versus Ag/AgCl reference electrode (*N* = 16). (**G**) *Z* score temporal evolution over 10 hours of electrical stimulation with 500-ms-long and −100-mV voltage pulses at 1 Hz (*V*_B_ = −0.3 V, *N* = 16, and *t*_ED_ = 45 s). (**H**) Box chart summarizing the influence of OCEAN geometry and operating voltage on time constants (*N* = 6).

For the rest of the electro-optic characterization, a total internal reflection–based illumination (Fig. 4A, inset, and section S4.2) was preferred over a dark-field condenser to confine the incident light to the OCEAN’s plane and minimize potential scattering background coming from the cells during biological experiments. An illumination wavelength of 637 nm was chosen as a trade-off between the OCEAN’s relative irradiance spectrum and the camera’s quantum efficiency. In addition, the variation of scattered light intensity for each OCEAN was expressed in terms of *Z* score. It represents how much the optical signal deviates from its baseline in terms of SDs and enables systematic comparison between OCEANs. Additional information about *Z* score computation can be found in Materials and Methods.

Figure 4A shows optical (top) and electrical (bottom) voltammograms acquired from OCEANs of different diameters in PBS. The optical signal scattered by each individual OCEAN was collected at the single-unit level. The OCEAN brightness follows a sigmoidal relationship with respect to bias voltages with a maximum slope between −0.3 and −0.4 V (see movie S1 for direct visualization of OCEAN electro-optic modulation during cyclic voltammetry). In addition, larger structures show stronger electro-optic responses accompanied by larger currents (see fig. S9, D to F, for dark-field images of arrays comprising OCEANs of different diameters). Voltammograms comprising two cycles are shown in section S4.3. As expected from our theoretical model, both voltage biases and cap diameters play a central role in the electro-optic characteristics of OCEANs. These contributions were systematically characterized by applying 1-s-long voltage pulses with amplitudes ranging from −100 to 100 mV across OCEANs with diameters of 0.7, 1.4, and 1.8 μm and operating at voltage biases between 0 and −0.6 V while monitoring their optical responses in PBS. Figure 4B shows representative optical traces measured from 1.4-μm-diameter OCEANs operating at a bias voltage *V*_B_ = −0.3 V. As anticipated by our theoretical model, a negative voltage pulse increased the OCEAN brightness, confirming enhanced PEDOT:PSS scattering properties in dedoped states. The overall dataset for the three different dimensions and the seven biases tested can be found in section S4.4. Figure 4 (C to F) and table S2 summarize the influence of geometries and operating biases on single OCEAN sensitivity, optical noise, SNR, and limit of detection. Predictably, the lowest limits of detection were consistently achieved within the bias voltage range of −0.3 to −0.4 V. The optimal operating voltages were −0.4 V for cap diameters of 0.7 and 1.8 μm and −0.3 V for 1.4-μm-diameter structures (see table S2 for limit of detection numerical values). In addition, the smallest OCEANs exhibited the most limited electro-optic responses. Even though higher sensitivity was observed for larger structures, the associated optical noise increase made the OCEANs of intermediate dimensions (*t*_ED_ = 45 s) perform similarly compared to the large ones (*t*_ED_ = 60 s) with a limit of detection between 2 and 3 mV at optimal bias.

The dynamic characteristics of OCEANs were experimentally investigated and are presented in Fig. 4H and section S4.5. Because of their larger capacitance, large OCEANs showed longer time constants than their smaller counterparts (Fig. 4H and fig. S13). At optimal bias, time constants were measured to be 6.0, 34.7, and 233.9 ms (mean values) for cap diameters of 0.7, 1.4, and 1.8 μm, respectively. Furthermore, negative voltage biases were associated with longer time constants, particularly for structures of intermediate dimensions. The increase in the serial resistance *R*_PEDOT:PSS_ caused by the dedoping of PEDOT at negative voltages (fig. S6) is likely responsible for this observation as the time constant is defined as $\tau =\left(R_{\text{PEDOT}:\text{PSS}}+R_{s}\right)\cdot C_{\text{PEDOT}:\text{PSS}}$ (see section S3 for additional information).

Last, the long-term stability of the OCEAN electro-optic properties was studied and is discussed in detail in section S4.6. As demonstrated in Fig. 4G, single OCEANs enabled wireless detection potentials of −100 mV for a continuous duration of 10 hours. While the *Z* score median value remained quite steady over the course of the experiment, its variation between OCEANs, as well as the number of outliers, became larger. Furthermore, the dynamic characteristics of several OCEANs seem to have considerably slowed down, as if PEDOT was steadily getting dedoped. The contribution of these affected OCEANs led to an overall SNR that decreased over time (fig. S14). The electro-optic characteristics of PEDOT:PSS OCEANs were not altered by 3 days of passive immersion in PBS at 37°C without electrical stimulation.

## DISCUSSION

OCEANs stand out by their fundamental mechanism that converts voltage fluctuations in an aqueous electrolyte into light variations, setting them apart from current state-of-the-art methods. For instance, OEM-based plasmonic antennas rely on their high charge density when highly doped (metallic state) to emit light through plasmonic resonance. By removing charges from the organic polymer through electrochemical dedoping processes, the brightness of the plasmonic antennas is quenched. The plasmonic resonance wavelength of PEDOT:PSS nanoantennas—defined by its geometry, complex permittivity, and the permittivity of the surrounding medium—is typically limited to the infrared window, which hinders experiments on regular biomicroscopy setups. Conversely, OCEANs leverage the dielectric state (low charge density) of dedoped PEDOT:PSS to emit light through scattering in the visible domain. The maxima in PEDOT:PSS real dielectric permittivity near the 700-nm wavelengths lead to bright scattering characteristics. By injecting charges in PEDOT:PSS through electrochemical doping processes, the OCEAN scattering is quenched. This fundamental principle difference enabled OCEANs to perform efficiently in the visible domain, where current biomicroscopy setups are optimal.

To achieve satisfactory SNRs, optical signals originating from multiple polymer plasmonic and electroplasmonic antennas are typically pooled together, hindering their inherent spatial resolution and number of recording sites in the context of biosensing. These features are also limited in electrode and OECT-based sensors because of the conductive traces connecting each sensing unit to an electrical instrument. Conversely, OCEANs enable wireless probing of potentials with high SNRs at the single-antenna level, eliminating the need to pool contributions from multiple sites and for individual conductive traces. Consequently, they permit unprecedented spatial resolution and an outstanding number of recording sites compared to existing biosensing technologies. Notably, the pitch between two OCEANs in an array was set to 5 μm throughout this work (fig. S9, D to F), theoretically leading to 4 million OCEANs on a 1 by 1 cm^2^ area. When using a 60× or 40× objective on a microscope with a 25-mm-diameter field of view (417 and 625 μm in diameter on the sample, respectively), we estimate that ~5000 or ~12,000 OCEANs, respectively, could be imaged simultaneously. Prospectively, this number could be enhanced by decreasing the pitch between OCEANs. Simultaneous multisite imaging is a considerable advantage of OCEANs compared to absorbance-based technologies, such as ECORE, which can only monitor a single recording site. Table S3 in the Supplementary Materials summarizes the electro-optic performance characteristics of OCEANs compared to state-of-the-art voltage-to-light transducers.

The theoretical model presented in this work describes how intermediate voltage biases drive PEDOT:PSS intermediate doping levels in PBS and how its complex permittivity is affected. These findings enabled us to accurately predict the electrochemical modulation of OCEAN scattering spectra and anticipate the central role of cap diameters and operating bias voltages on antenna brightness and sensitivity. While larger structures were shown to be brighter and more sensitive than their smaller counterparts, their time constant was also predicted to be slower following the systematic electrochemical characterization of the OCEAN-electrolyte interface by electrochemical impedance spectroscopy. Because of this trade-off between electro-optic modulation and dynamic performances, antennas with small, intermediate, and large diameters were fabricated to determine an optimal dimension experimentally.

The nanofabrication process developed to manufacture OCEANs was reliable and led to homogeneous structures across fabrication batches. The FIB lithography step was performed using a Velion FIB (Raith Nanofabrication, Germany). Compared to traditional FIBs, the Velion FIB was designed for large-scale nanofabrication. It comprises a laser-interferometric stage enabling stitching with a resolution of 1 nm and, therefore, permitting nanoscale patterning across large areas. In addition, its vertical ionic column uses gold double-plus ions (Au^++^), whose sputtering yield is higher than that of gallium ions, therefore minimizing the patterning time. These characteristics enhanced the scalability of our process. While using the Velion FIB proved convenient for rapid design iterations, it is not essential to successfully fabricate OCEANs. Any conventional (e.g., electron-beam or deep ultraviolet lithography) or alternative (e.g., scanning probe lithography) nanofabrication technique capable of patterning 250-nm hole arrays in a 50-nm-thick layer of silicon nitride would be suitable for manufacturing OCEANs, ensuring broad accessibility to the proposed technology. For example, using a stepper, 250-nm-diameter openings could be patterned in a photoresist layer at the wafer scale and subsequently transferred into the silicon nitride by reactive ion etching. This approach could offer a high-throughput manufacturing solution for OCEANs, considerably reducing the cost per chip. The optical monitoring of PEDOT:PSS electrodeposition in real time enabled compensating for potential variability in the process and was pivotal in establishing consistency between batches.

The total internal reflection dark-field microscopy setup we developed enabled the selective collection of the light scattered by OCEANs over the incident light. Compared to conventional dark-field microscopy, this approach minimizes the background scattering—which could come from the cell culture—and ensures a consistent translation of OCEAN electro-optic characteristics to biological experiments. In addition, compared to methods using single photodetectors, it enables the formation of an image of the array with submicrometer resolution, providing spatial information. The illumination wavelength of 637 nm was selected not only to maximize OCEAN electro-optic characteristics but also to minimize potential adverse effects on cells. In addition to phototoxicity, the photobleaching of pharmacological compounds at shorter wavelengths is a well-known issue that could have hindered the applications of this technology. For instance, blebbistatin—a cardiomyocyte contraction inhibitor widely used to prevent mechanical artifacts with optical electrophysiology techniques—is known to photobleach at wavelengths below 500 nm (*40*). Hence, using a broadband light source or a blue-shifted illumination would inhibit the blebbistatin effect and render electrophysiological studies with cardiomyocytes irrelevant.

The systematic experimental electro-optic characterization of OCEANs demonstrated that single units could transduce potential fluctuations into light variations with SNRs of 48 for 100-mV voltage pulses and limits of detection of 2.5 mV. The theoretically predicted relationships linking operating voltage biases and cap diameters to the antennas’ brightness and sensitivity are consistent with our experimental observations. In particular, the fact that positive voltage pulses led to negative variations of the OCEAN optical signal demonstrates that the fundamental mechanism governing OCEANs is not based on absorbance—where a positive voltage pulse would induce a positive fluctuation of the optical signal—but instead relies on the modulation of the scattering properties of the device. The relationship between the scattering signal and voltage is linear near the optimal operating voltage bias. A systematic comparison between the electro-optic performance of OCEANs and state-of-the-art voltage-to-light transducers is difficult as experimental conditions and outcomes are rarely comparable. For instance, ECORE showed an enhanced limit of detection compared to individual OCEANs but with illumination intensity orders of magnitude higher (*25*). Single antennas exhibited an improved limit of detection compared to arrays of electroplasmonic nanoantennas in physiological solution under reasonably equivalent experimental conditions [estimated from figure 4B of (*29*)]. Nevertheless, potential strategies to improve the limit of detection of OCEANs should be explored, including optimizing the optical setup and exploring materials with enhanced sensitivity. The optical setup-OCEAN system as a whole defines the detection limit. In particular, the optical setup’s mechanical stability should be prioritized, as methods relying on scattering with coherent light sources are naturally prone to speckle formation resulting from partial reflections at optical elements (*41*). This background interference pattern can be quickly altered by changes in the optical path because of temperature fluctuations, laser wavelength drift, or mechanical instability and was identified as the primary source of noise in our experiments. Enhancing the optical setup mechanical stability through passive or active mechanisms (*41*) can potentially improve OCEAN’s detection limit. Other polymers and treatments optimized in the context of OECTs should be investigated for material optimization (*42*). Alternatively, where bandwidth is not a limiting factor, increasing the antenna diameter beyond 1.8 μm could potentially enhance OCEANs’ sensitivity and limit of detection, as long as the noise could remain controlled.

Furthermore, experimental measurements of OCEAN dynamic characteristics agree with our theoretical predictions of time constants ranging from a few milliseconds to more than a hundred milliseconds at optimal biases for small and large structures, respectively. Currently, OCEAN time responses are mostly limited by large values of *R*_PEDOT:PSS_, mainly imposed by the small stem diameter of the PEDOT:PSS structure. Prospectively, faster dynamic characteristics could be achieved by modifying the OCEAN geometry from a mushroom-like to disk-like structure of the same volume but with an augmented contact area with the ITO electrode. This way, the volumetric PEDOT:PSS capacitance would remain constant and *R*_PEDOT:PSS_ be reduced, thus enabling faster time constants.

Last, OCEANs demonstrated exceptional long-term characteristics with continuous electro-optic modulation capabilities for at least 10 hours. This long-term stability is a distinct advantage over fluorescence-based indicators (e.g., voltage-sensitive dyes or genetically encoded voltage indicators), which photobleach within minutes. It demonstrates the potential of OCEANs to perform biosensing studies over time.

As a case study, we developed a Luo-Rudy–derived analytical model of the cell-OCEAN interface to investigate the feasibility of recording intracellular cardiomyocyte action potentials using OCEANs (section S5). The model’s outcome suggests that even for a conservative estimate of the seal and junctional resistance *R*_Seal_ = 200 megohms and *R*_j_ = 500 megohms (*43*, *44*), respectively, a considerable voltage drop of 30 mV—far above the detection limit of OCEANs—is expected across the PEDOT:PSS structure, demonstrating the feasibility of using OCEANs for intracellular electrophysiological studies. In addition, theoretical modeling of OCEAN electro-optic scattering properties in the presence of cells revealed minimal scattering contribution from cells under total internal reflection illumination, indicating that similar electro-optic performance can be expected during biosensing applications (section S5.4). Compared to state-of-the-art plasmonic antenna arrays and absorbance-based technologies, OCEANs protrude from an otherwise insulating substrate. This ensures an enhanced seal resistance at the cell-OCEAN interface and enables intracellular access following electroporation, maximizing the electrophysiological signal amplitude transferred to the sensor. The next iteration of OCEANs could have their stem exposed—similar to mushroom-like microelectrodes—to leverage cell engulfment and reinforce their interface with the cell (*45*–*47*). Besides demonstrating feasibility, the presented model illustrates fundamental aspects to consider when using OCEANs for biosensing compared to conventional electrode-based sensors (section S5.1). In this context, OCEANs have the potential to enhance both the spatial resolution compared to multielectrode arrays and the long-term stability compared to fluorescent reporters, thus potentially opening new opportunities in electrophysiology. As a next step, proof-of-concept electrophysiological recordings from electrogenic cell networks should be performed to demonstrate the feasibility of OCEANs experimentally. Cardiomyocyte monolayers should be used because of their cell-electrode interface reliability and their electrical activity synchronicity. Hence, integrating planar microelectrodes with OCEAN arrays could provide simultaneous ground truth signals. OCEANs of various geometries could be explored to enhance the cell-OCEAN interface and improve the quality of the recordings.

In summary, we introduced the concept of OCEANs and investigated their feasibility to wirelessly probe electrical activity with high spatial resolution for biosensing applications. This technology leverages the intrinsic dependence of PEDOT:PSS scattering properties on its doping levels to transduce small potential fluctuations into variations of scattered light intensity in the visible spectral window. We developed a theoretical model describing the relationship between voltage, doping level, and complex permittivity in PEDOT:PSS to predict the scattering properties of single OCEANs with varying dimensions and operating at different voltage biases in response to electrical stimulation. A reliable nanofabrication process combining next-generation FIB lithography with conventional microfabrication techniques was established to precisely manufacture arrays of OCEANs with diameters as low as 0.7 μm. The electro-optic properties of OCEANs were systematically characterized by applying various electrical stimuli in PBS while monitoring scattered light intensity using a custom-designed total internal reflection dark-field microscope. Single OCEANs showed SNRs up to 48 in response to a 100-mV voltage pulse with a limit of detection of 2.5 mV at optimal operating biases. Time constants ranged between 6.0 and 233.9 ms for structures of 0.7 and 1.8 μm, respectively. OCEANs also demonstrated exceptional long-term stability, enabling continuous electro-optic modulation for 10 hours. Last, an analytical cell-OCEAN model derived from Luo-Rudy was implemented to demonstrate the feasibility of recording cardiomyocyte intracellular action potentials using OCEANs.

OCEANs can potentially enable functional readout studies from thousands of recording sites simultaneously, with micrometer spatial resolution, and over extended periods of time. Such recording characteristics make OCEANs a great candidate to overcome the technical limitations of current biosensing approaches, opening new opportunities in bioelectronics and, prospectively, accelerating progress in both fundamental and clinical research.

## MATERIALS AND METHODS

### Modeling of OCEANs

The scattering cross sections of PEDOT:PSS OCEANs were simulated using the finite element modeling method provided by CST Studio Suite software (Dassault Systemes, France). The OCEAN stem diameter (SiN*_x_* opening) was set to 250 nm. Two cap diameters, 750 nm and 1.5 μm, were investigated for which substrate lateral dimensions of 1000 and 1750 nm were defined, respectively. The SiN*_x_*, ITO, and glass substrate thicknesses were set to 50, 70, and 200 nm, respectively. Water was defined as the background material. Perfectly matched layer boundary conditions were used in all directions of the space, with extra space added in the upward vertical (+*z*) direction to ensure the reliability of the far-field computation. The frequency-dependent permittivities of glass (*48*) and ITO (*49*) were extracted from publicly available datasets, while constant permittivity values of 4 and 1.77 were used for SiN*_x_* (*50*) and water (*51*), respectively. PEDOT:PSS complex permittivities computed for different voltage biases (section S1) were fed into the model to account for electrochemical modulation. A plane wave with an electric field pointing in the *x* direction and propagating downward (−*z* direction) was used as excitation. Spatial symmetries of the simulated structure were leveraged to shorten the simulation time without compromising the accuracy of the simulation (*yz* plane defined as the electric field symmetry plane and *xz* plane defined as the magnetic field symmetry plane). Scattering cross sections were simulated for all directions of space (angular resolution of 1°) and wavelength ranging from 400 to 1000 nm (with 10-nm increments) using the frequency domain solver. Angular integration of the three-dimensionally (3D) resolved scattering radiation patterns was performed using MATLAB (MathWorks, USA), from which the total scattering cross section of our OCEANs for all simulated wavelengths and voltage biases could be extracted.

To estimate the dynamic response of an OCEAN at a specific wavelength (Fig. 2H), the voltage trace across PEDOT:PSS in response to a 100-mV voltage pulse was computed using the electrical equivalent circuit presented in fig. S6A (assuming ideal resistance-capacitance–type behavior). Impedance characteristics of OCEANs with diameters of 0.75 and 1.5 μm were estimated from experimental measurements of OCEANs of 0.7 and 1.4 μm, respectively. Scattering cross sections corresponding to the voltage levels anticipated at different time points were interpolated from the simulated scattering cross section using a sigmoid characteristic to determine the OCEAN optical response.

The influence of the cell presence on OCEAN scattering properties was evaluated under total internal reflection illumination conditions (incidence angle of 70° with respect to the vertical axis), collecting the scattered light from the top. The cell membrane thickness was set to 5 nm, and 20 nm was placed on top of the antenna. The cytosol thickness was set to 100 nm with perfectly matched layer boundary conditions in all directions of the space. Refractive indexes of 1.54 and 1.38 were used to simulate the cell membrane (*52*) and the cytoplasm (*53*), respectively. The dedicated data repository contains CST and MATLAB files and any other supplementary materials necessary to reproduce the presented model (see Data and materials availability in Acknowledgments).

### Nanofabrication and interfacing of OCEANs

ITO-coated glass substrates (dimensions, 22 by 22 mm^2^; thickness, 1.5 to 170 μm; resistivity, 70 to 100 ohms/□; from SPI Supplies, USA) were subsequently sonicated for 5 min in deionized water and isopropyl alcohol. A multimeter was used to identify the side of the chips presenting the ITO layer, and their orientation was carefully maintained throughout the fabrication process (step 1, fig. S3).

#### ITO patterning and passivation

For ITO patterning and passivation (steps 2 and 3, fig. S3), the substrates underwent a 300-W, 0.8-mbar, and 2-min-long oxygen plasma step (Nano-QL-PCCE7, Diener Electronic, Germany) and functionalization with hexamethyldisilazane for 10 s at 150°C in a YES-310TA vacuum oven (Yield Engineering Systems, USA) in preparation for photolithography. A 1.8-μm-thick AZ3310 positive photoresist layer (MicroChemicals, Germany) was spin coated at 1500 rpm for 60 s using a CEE 200X-F spin coater (Brewer Science, USA) and baked at 90°C for 60 s. The photoresist was exposed using an MLA-150 maskless aligner (Heidelberg, Germany) with a dose of 600 mJ/cm^2^, baked at 110°C for 60 s, and developed for 30 s in AZMIF300 (MicroChemicals, Germany) to define the traces and pads of the devices. A 15-min-long hard-bake step at 95°C was performed to ensure the maximal stability of the photoresist mask during the subsequent etching step. The ITO layer was patterned by a 5-min-long chlorine-based reactive-ion etching step using a 230i inductively coupled plasma reactive ion etcher (Samco, Japan). The photoresist was removed by subsequent steps of oxygen plasma ashing (300 W, 0.8 mbar, 2 min, Nano-QL-PCCE7, Diener Electronic, Germany) and sonication in *N*-methyl-2-pyrrolidone for 1 hour and 30 min. The resulting ITO traces and pads were passivated by a 50-nm-thick layer of SiN*_x_* deposited by plasma-enhanced chemical vapor deposition performed in a PD220NL deposition tool (Samco, Japan) using silane and ammonia and validated with an F20 thin-film measurement system (Filmetrics, USA).

#### SU8 patterning

For SU8 patterning (step 4, fig. S3), the SiN*_x_*-coated patterned substrates underwent a 300-W, 0.8-mbar, and 2-min-long oxygen plasma step (Nano-QL-PCCE7, Diener Electronic, Germany) and functionalization with hexamethyldisilazane for 10 s at 150°C in a YES-310TA vacuum oven (Yield Engineering Systems, USA) in preparation for photolithography. An Apogee spin coater (Cost Effective Equipment, USA) was used to spin coat a 3-μm-thick layer of GMPI1040 SU8 (Gersteltec, Switzerland) at 1100 rpm for 62 s (11 s of acceleration and deceleration at 100 rpm/s and 40 s of steady rotation at 1100 rpm). After 5 min of relaxation, the substrates were baked for 5 min at 65°C and 10 min at 95°C. An MLA 150 maskless aligner (Heidelberg, Germany) was used to expose the negative SU8 photoresist at a dose of 1200 mJ/cm^2^. Following exposure, postexposure baking was performed (5 min at 65°C and 10 min at 95°C), followed by a 10-min-long rehydration delay. SU8 was manually developed by immersion in propylene glycol methyl ether acetate for 40 s and hard-baked at 135°C for 2 hours.

#### SiN_x_ patterning

For SiN*_x_* patterning (step 5, fig. S3), a Velion FIB (Raith Nanofabrication, Germany) was used to pattern 250-nm-diameter openings in the exposed SiN*_x_* layer. High-sputtering yield Au^++^ were used to maximize the patterning throughput, and a 10-μm aperture was set to maintain a beam current of ~9 pA, enabling fine spatial resolution. The optimal patterning dose was found to be 0.2 nC/μm^2^ distributed across 10 outward loops (0.02 nC/μm^2^ per loop).

#### Interfacing

For interfacing, H20E conductive epoxy (Epotek, USA) was manually dispensed on the pads of a custom-designed PCB using a precision fluid dispenser (APPLSRA105-10CC, SRA Soldering Products, USA). Subsequently, the chip was accurately positioned and pressed onto the PCB (setpoint of 10 g) using an M-3 pick and place tool and cured at 80°C for 3 hours. Then, insulating epoxy 302-3M (Epotek, USA) was manually applied at the interface between the chip and the PCB before being baked at 65°C for 3 hours to render the interface mechanically stable and waterproof. Last, a 6-mm-high glass ring (36-mm outer diameter and 32-mm inner diameter, Friedrich and Dimmock, USA) was assembled using the 302-3M epoxy and baked for 3 hours at 65°C to finalize the interfacing.

#### PEDOT:PSS electrodeposition

For PEDOT:PSS electrodeposition (step 6, fig. S3), to make the chip hydrophilic and prevent air bubbles from being trapped in the chip cavities, a 30-W, 30-s, and 0.6-mbar oxygen plasma treatment was performed using an Atto plasma asher (Diener Electronic, Germany). Subsequently, an electrochemical cleaning step was performed by sweeping the potentials between the ITO layer and a 50 mM solution of potassium hydroxide from −0.2 to −0.8 V versus Ag/AgCl for at least 10 cycles using a three-electrode setup and a Compactstat potentiostat (Ivium, The Netherlands). A platinum wire was used as the counter electrode, and an RE-1B Ag/AgCl reference electrode (ALS-japan, Japan) was used as the reference. The solution used to electrodeposit PEDOT:PSS was composed of 10 mM 3,4-ethylenedioxythiophene (483028-10G, Sigma-Aldrich, USA) and 0.1 M poly(sodium 4-styrene sulfonate) (NaPSS, 243051-100G, Sigma-Aldrich, USA). Note that 0.1 M NaPSS corresponds to the concentration of sodium 4-styrene sulfonate monomers (206 mg of NaPSS in 10 ml of deionized water). PEDOT:PSS OCEANs were electrodeposited by applying 0.9 V versus Ag/AgCl for 30, 45, or 60 s using the same three-electrode setup.

### Electrochemical impedance spectroscopy on OCEANs

Electrochemical impedance spectroscopy of OCEANs was performed in PBS (806552-500ML, Sigma-Aldrich, USA) using a Compactstat potentiostat (Ivium, The Netherlands) in a three-electrode configuration. A 10-mV-amplitude sinusoidal voltage signal with frequencies ranging between 1 and 100,000 Hz was applied between the ITO and the reference electrode of individual arrays. The fitting of the experimental data was directly performed using the Ivium software.

Raw EIS data and MATLAB codes used to postprocess and plot the electrochemical characteristics of OCEANs are available in the dedicated data repository (section S6).

### Electro-optic characterization of OCEANs

#### Scattering spectral acquisition

Scattering spectra of PEDOT:PSS OCEAN arrays (50 by 50 OCEANs, ~1.8 μm in diameter) were acquired in PBS using a Ti2E inverted microscope (Nikon, Japan) configured with a dark-field condenser to enable dark-field microscopy. An SLS302 broadband light source (Thorlabs, USA) was connected to the microscope via an LLG3-4Z liquid guide (Thorlabs, USA) and used for diascopic illumination. A 40× objective was used to collect the light scattered by the OCEANs, which was subsequently transferred to an Ocean-HDX-XR spectrometer (Ocean Insight, USA) through a 1000-μm optical fiber (QP1000-2-VIS-NIR, Ocean Insight, USA). The spectrometer slit was set to 100 μm. The OCEAN spectra were subtracted by the one acquired under dark conditions and normalized by the light source spectrum in the relative irradiance method of the spectrometer Ocean View software (Ocean Insight, USA). The raw spectra and the MATLAB code implemented to postprocess them are available in the dedicated data repository (section S6).

#### Total internal reflection dark-field microscope setup

A custom-designed upright wide-field fluorescence microscope body was built from the DIY Cerna components available at Thorlabs, USA. A Chrolis-C1 (Thorlabs, USA) multiwavelength light engine was used as the episcopic light source for fluorescence and bright-field microscopy. An Orca Fusion-BT (Hamamatsu, USA) camera was selected as the readout sensor for fluorescence, bright-field, and dark-field microscopy. A water dipping 60× objective (N60X-NIR, Thorlabs, USA) was used for the entire electro-optic characterization of OCEANs. A prism-based total internal reflection illumination module was built using a 637-nm pig-tailed laser diode (70 mW, LP637-SF70, Thorlabs, USA) mounted on a CLD1010LP controller (Thorlabs, USA), and its output collimated with an F230FC-B collimator (Thorlabs, USA). The collimator was mounted at 30° with respect to the horizontal plane on an M30XY/M motorized stage (Thorlabs, USA) and aligned with an ADB-10 prism (Thorlabs, USA). The entire illumination module was assembled on an L490/M lab jack (Thorlabs, USA) for coarse *Z* adjustments. A custom 3D-printed sample holder was assembled on an MPRC/M recording chamber holder (Thorlabs, USA) and fixed on an MP20 rigid stand mounted on a two-dimensional motorized translation stage (PLS-XY, Thorlabs, USA) to enable accurate motion of the sample.

#### Electro-optic characterization of OCEANs

A Compactstat potentiostat (Ivium, The Netherlands) was used in a three-electrode configuration to apply electrical stimuli (e.g., cyclic voltammetry and chronoamperometry) to OCEANs in PBS. A platinum wire was used as the counter electrode, and an RE-1B Ag/AgCl reference electrode (ALS-japan, Japan) was used as the reference. The camera exposure time and frame rate were set to 1 ms and 200 frames per second (fps) for chronoamperometry and 1 ms and 20 fps for cyclic voltammetry, respectively. For time-constant measurements, the exposure time was set to 200 μs and the frame rate to 2000 fps. The sensitivity of each OCEAN was estimated by performing a linear fit on its optical *Z* score response to voltage pulses of varying amplitudes (figs. S10H, S11H, and S12H). The sensitivity, represented by the slope of this fit, quantifies the change in the *Z* score per unit of applied voltage ($\text{Sensitivity}=\frac{∆Z}{∆V}$, where ∆Z is the variation in the optical *Z* score in response to a voltage change ∆V based on the linear interpolation of experimental data). The noise was defined as the SD of the *Z* score over a 1-s period in the absence of stimuli ($\text{Noise}=\sqrt{\frac{1}{N}\sum_{i=1}^{N}{\left(Z_{i}-\bar{Z}\right)}^{2}}$, where $Z_{i}$ represents the *Z* score at each time point, $\bar{Z}$ is the mean *Z* score during the 1-s interval, and *N* is the total number of time points recorded). The SNR was calculated by dividing the *Z* score response of each OCEAN to a −100-mV voltage pulse by its corresponding noise level ($\text{SNR}=\frac{Z_{100\text{mV}}}{\text{Noise}}$, where $Z_{100\text{mV}}$ is the *Z* score in response to the −100-mV voltage pulse). Last, the limit of detection was defined as the voltage for which the SNR was equal to 1. Practically, it was computed by finding the intersection between the linear relationship describing the *Z* score versus voltage stimulus amplitudes and the noise level for each OCEAN ($\text{SNR}=1=\frac{\text{Sensitivity}\cdot V_{\text{LOD}}}{\text{Noise}}⟺V_{\text{LOD}}=\frac{\text{Noise}}{\text{Sensitivity}}$, where $V_{\text{LOD}}$ is the limit of detection).

#### Data analysis

Image sequences were first processed with a vibration compensation algorithm (*54*) to prevent motion artifacts from biasing the electro-optic characterization. Circular regions of interest (ROIs) with a radius of 20 pixels were defined around individual OCEANs. For each pixel of the ROIs, the average ($\mu_{\text{Pix}}$) and SD ($\sigma_{\text{Pix}}$) of the optical signal in the absence of electrical stimulation were computed and used to calculate a *Z* score per pixel [$Z\;{\text{score}}_{\text{Pix}}\left(t\right)=\frac{X_{\text{Pix}}\left(t\right)-\mu_{\text{Pix}}}{\sigma_{\text{Pix}}}$, with $X_{\text{Pix}}\left(t\right)$ being the raw optical signal from a single pixel] for each time point. Pixels with a *Z* score variation larger than 3 were considered part of the OCEAN and averaged to define its optical response. Electro-optic characterization results were summarized in box charts displaying the median, lower, and upper quartiles, outliers, and the minimum and maximum values that are not outliers (MATLAB, USA).

The dedicated data repository contains raw image sequences, MATLAB codes, and any supplementary material implemented or collected during the electro-optic characterization of OCEANs.

### Modeling of cardiomyocyte action potential recording with OCEANs

The cell-OCEAN interface model was adapted from a MATLAB implementation of the Luo-Rudy model (GitHub permalink: https://github.com/meeravarshneya1234/ventricular_myocyte_models/tree/a57aaf7a632b3808fe0fe93355c2a8d9eed16dd0/Luo_Rudy91_Model). The updated code is available in the dedicated data repository. Equations, numerical values of the key components used in the model, and additional discussions related to its implementation are presented in section S5.

## Supplementary Materials

### The PDF file includes:

Supplementary Text Figs. S1 to S16 Tables S1 to S4 Legend for movie S1 References
