# Supplementary material for: Organic electro-scattering antenna: Wireless and multisite probing of electrical potentials with high spatial resolution
Source: Sci Adv. 2024 Dec 20;10(51):eadr8380. doi: 10.1126/sciadv.adr8380 (PMC11661451; doi:10.1126/sciadv.adr8380)
Supplement: Supplementary file 1 — Supplementary Text Figs. S1 to S16 Tables S1 to S4 Legend for movie S1 References [file sciadv.adr8380_sm.pdf]

Science Advances
AAAS

Supplementary Materials for

Organic electro-scattering antenna: Wireless and multisite probing of electrical potentials with high spatial resolution

Benoit Desbiolles *et al.*

Corresponding author: Deblina Sarkar, deblina@mit.edu

Sci. Adv. **10**, eadr8380 (2024)
DOI: 10.1126/sciadv.adr8380

The PDF file includes:

Supplementary Text
Figs. S1 to S16
Tables S1 to S4
Legend for movie S1
References

Other Supplementary Material for this manuscript includes the following:

Movie S1

1 Electrochemical doping and dedoping of PEDOT:PSS

In this section, the doping and dedoping processes taking place within the PEDOT backbone are first discussed. Subsequently, the PEDOT:PSS complex permittivity is presented for different doping levels (Sub-section 1.2) and voltages (Sub-section 1.3).

1.1 Doped and dedoped states of PEDOT:PSS

Figure S1 illustrates how the PEDOT doping level is modulated by external voltage biases across the PEDOT:PSS-electrolyte interface. At equilibrium, holes (*i.e.,* polarons and bipolarons) in the PEDOT backbone are stabilized by the fixed sulfonate anions in the PSS strand. Under negative voltage, solvated cations from the electrolyte neutralize the negatively charged PSS strands and locally reduce the PEDOT into its dedoped state. The resulting hole is extracted through the indium tin oxide (ITO) electrode. Conversely, injecting holes under positive voltage biases across the interface can oxidize the PEDOT backbone into its doped state.(*37*) In response to electrochemical doping/dedoping processes, structural changes in the PEDOT backbone directly modulate the optical absorbance and scattering properties of the film, which are naturally captured in the complex (frequency-dependent) refractive index denoted $m = n + i\kappa$.

The real part of the refractive index n indicates the phase velocity of light in the material and the imaginary part κ , which represents the material's extinction coefficient. In light scattering theory, the real refractive index n is directly associated with a particle's scattering cross-section – defined as the ratio between the scattered light's power and the incident light's intensity – and modulates its scattering characteristics.(*40*) A material's complex refractive index can be expressed in terms of its complex permittivity $\varepsilon = \varepsilon_1 + i\varepsilon_2$, where $\varepsilon_1 = n^2 - \kappa^2$ and $\varepsilon_2 = 2n\kappa$.(*56*) For low κ values, ε_1 tends to n^2 and ε_2 to zero.

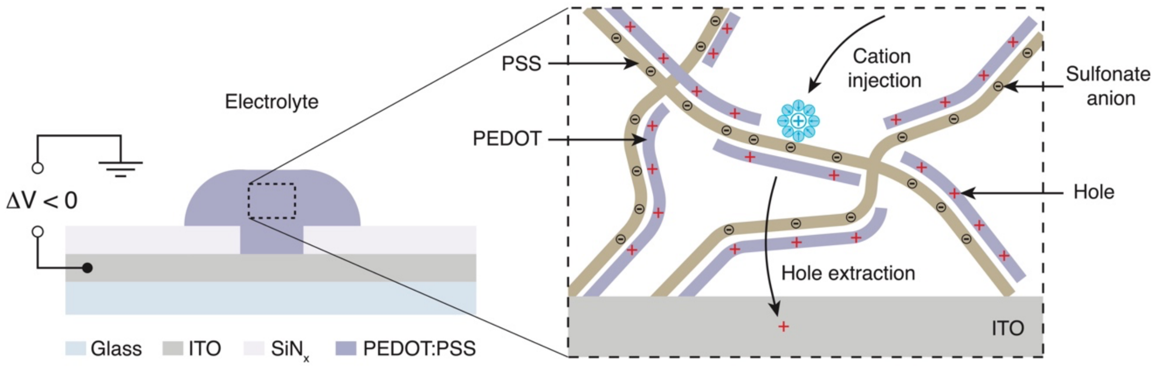

Figure S1: Doping and dedoping of PEDOT:PSS. Illustration showing the PEDOT:PSS dedoping process when a negative voltage bias is applied across the organic electro-scattering antenna-electrolyte interface. Adapted with permission from Proctor *et al.*(*37*)

1.2 Relationship between PEDOT:PSS doping level and permittivity

The complex permittivities of PEDOT:PSS in its fully doped and fully dedoped states were fitted from a recent *in-situ* spectroscopic ellipsometry study(*27*) (Figure S2-A and B). We assume these extreme-state permittivities to be a universal property of the material and invariant with respect to the electrolyte in which they were measured. The transient increase in absorption occurring near 1000 nm for intermediate biases was neglected as its exact voltage onset is electrolyte-dependent and difficult to estimate accurately from the data provided.(*27*) The doped state complex permittivity is best described by a Drude model (red curves in Figure S2-A and B), which is consistent with a metallic behavior. The dedoped state complex permittivity is instead best fitted by a combination of two Lorentz and two Tauc-Lorentz functions, consistent with a purely dielectric behavior of the PEDOT:PSS in this regime (black curves in Figure S2-A and B, resulting from the combination of the four models depicted in dashed lines).

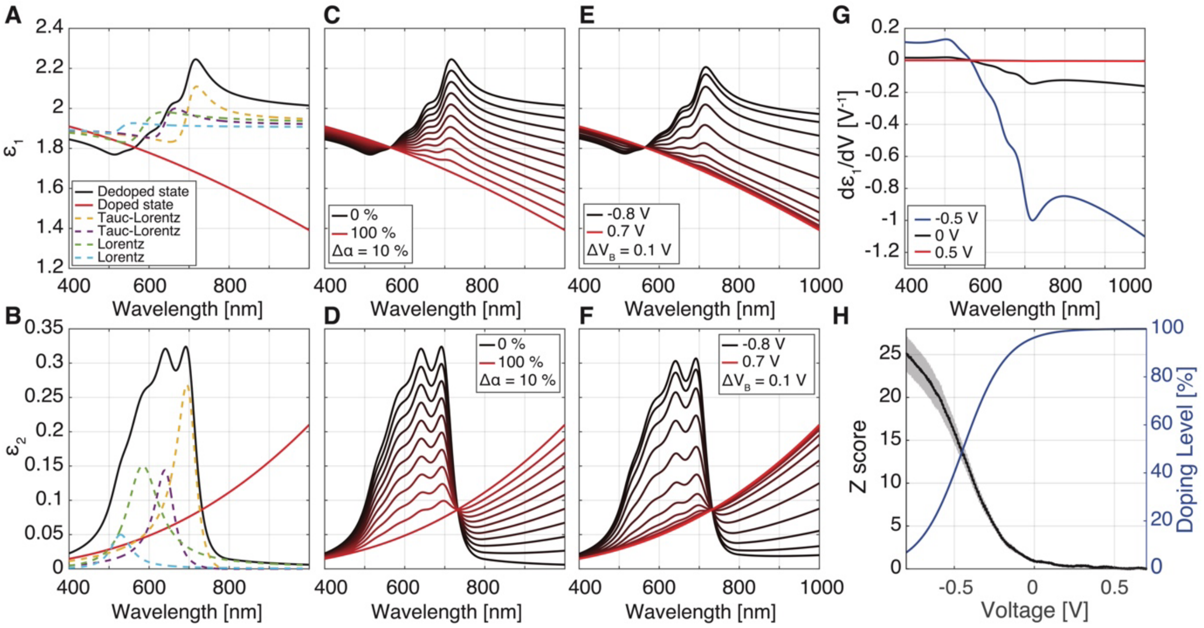

Figure S2: Electrochemical modulation of PEDOT:PSS complex permittivity. A) Real and B) imaginary part of the PEDOT:PSS complex permittivity in its fully doped (red curve) and fully undoped (black curve) state, fitted from reference(*27*). The fully doped state is fitted by a single Drude model, while the fully undoped state by a combination of two Lorentz and two Tauc-Lorentz models (dashed lines). C) Real and D) imaginary parts of the PEDOT:PSS complex permittivity for intermediate doping levels α. E) Real and F) imaginary parts of the PEDOT:PSS complex permittivity for different voltage bias levels V_B in phosphate-buffered saline (PBS) solution. G) Variations of the PEDOT:PSS real permittivity with respect to wavelength for different voltage bias levels. H) Relative variation of scattered light intensity (Z score) collected from individual PEDOT:PSS organic electro-scattering antennas in response to a linear voltage sweep from 0.7 V to -0.8 V in PBS vs Ag/AgCl (N=16, mean ± standard deviation). Assuming that the optical signal variations are solely caused by a variation of the PEDOT doping level, the link between voltage bias and doping level can be established by fitting the optical signal with a sigmoidal function.

The corresponding complex permittivity for the Drude(*57*) ($\varepsilon_D(\omega)$), Lorentz(*58*) ($\varepsilon_L(\omega)$), and Tauc-Lorentz(*59*) ($\varepsilon_{TL}(\omega)$) models are recapitulated below:

$$\varepsilon_D(\omega) = \varepsilon_\infty - \frac{{\omega_P}^2}{\omega(\omega + i\gamma)} \qquad \text{Equation S1}$$

$$\varepsilon_L(\omega) = \varepsilon_\infty + \frac{{\omega_P}^2}{{\omega_0}^2 - \omega^2 - i\Gamma\omega} \qquad \text{Equation S2}$$

$$\varepsilon_{TL}(\omega) = \varepsilon_\infty + \chi_{TL}(E) \qquad \text{Equation S3}$$

With $E = \frac{h\omega}{2\pi}$, $\Im\{\chi_{TL}(E)\} = \begin{cases} \frac{1}{E}\frac{AE_0C(E-E_g)^2}{(E^2-{E_0}^2)^2+C^2E^2}, & \text{if } E > E_g \\ 0, & \text{if } E \leq E_g \end{cases}$, and $\Re\{\chi_{TL}(E)\} = \frac{2}{\pi}\int_{E_g}^{\infty}\frac{\xi\Im\{\chi_{TL}(E)\}}{\xi^2-E^2}d\xi$

Where:

- ω is the angular frequency
- ε_∞ is the static dielectric constant
- ω_P is the plasma frequency
- γ is the characteristic collision frequency
- ω_0 is the resonance frequency of the oscillator
- τ is the relaxation time
- $\Gamma = \frac{1}{\tau}$ is the inverse of the relaxation time
- E is the photon energy
- $h = 6.626.10^{-34}J.Hz^{-1}$ is the Planck constant
- $\chi_{TL}(E)$ is related to the electric susceptibility
- A is a fitting parameter related to the strength of the Lorentzian oscillator
- E_0 is a fitting parameter related to the resonant frequency of the Lorentzian oscillator
- C is a fitting parameter related to the broadening of the Lorentzian oscillator
- E_g is a fitting parameter related to the bandgap material

The complex permittivity for the fully doped and fully dedoped states of PEDOT:PSS can be expressed as follows:

$$\varepsilon_{Doped}(\omega) = \varepsilon_D(\omega) \qquad \text{Equation S4}$$

$$\varepsilon_{Dedoped}(\omega) = \varepsilon_{L,1}(\omega) + \varepsilon_{L,2}(\omega) + \varepsilon_{TL,1}(\omega) + \varepsilon_{TL,2}(\omega) \qquad \text{Equation S5}$$

The values of the parameters fitted from the experimental data presented in reference(*27*) are summarized in Table S1.

Table S1: Numerical values used to model PEDOT:PSS complex permittivity. Values of the parameters of the Drude, Lorentz, and Tauc-Lorentz models contributing to the fully doped and fully dedoped complex permittivity of PEDOT:PSS, fitted from experimental data presented in reference(*27*).

Model	Parameter	Value	Unit
$\varepsilon_{\infty,Doped}$		2.02	-
ε_D	ω_P	$1.58 \cdot 10^{15}$	$rad \cdot s^{-1}$
	γ	$0.630 \cdot 10^{15}$	$rad \cdot s^{-1}$
$\varepsilon_{\infty,Dedoped}$		1.9	-
$\varepsilon_{L,1}$	ω_P	$0.512 \cdot 10^{15}$	$rad \cdot s^{-1}$
	ω_0	$3.24 \cdot 10^{15}$	$rad \cdot s^{-1}$
	Γ	$0.539 \cdot 10^{15}$	$rad \cdot s^{-1}$
$\varepsilon_{L,2}$	ω_P	$0.273 \cdot 10^{15}$	$rad \cdot s^{-1}$
	ω_0	$3.57 \cdot 10^{15}$	$rad \cdot s^{-1}$
	Γ	$0.419 \cdot 10^{15}$	$rad \cdot s^{-1}$
$\varepsilon_{TL,1}$	A	$3.90 \cdot 10^{-19}$	J
	E_0	$2.83 \cdot 10^{-19}$	J
	C	$2.34 \cdot 10^{-20}$	J
	E_g	$2.48 \cdot 10^{-19}$	J
$\varepsilon_{TL,2}$	A	$1.05 \cdot 10^{-19}$	J
	E_0	$3.09 \cdot 10^{-19}$	J
	C	$2.84 \cdot 10^{-20}$	J
	E_g	$2.48 \cdot 10^{-19}$	J

All the complex permittivities presented above exhibit a linear relationship with their respective number density (*i.e.,* the volumetric density of the charge carriers contributing to the permittivity) through the plasma frequency parameter $\omega_P{}^2$ in their numerators. Note that A is related to the strength of the Lorentzian oscillator inherent to the Tauc-Lorentz model, which is proportional to $\omega_P{}^2$.(*58*)

Indeed, $\omega_P{}^2$ is defined as:

$$
{\omega_p}^2 = \frac{e^2 N}{\varepsilon_0 m^*}
$$
Equation S6

Where e is the elementary charge, ε_0 is the vacuum permittivity, m^* is the effective mass of the charge carrier considered, and N is the volumetric density of charge carriers contributing to the permittivity. For ε_{Doped}, the PEDOT:PSS complex permittivity in its doped state, $N = N_{Doped}$ and represents the number density of polarons and bipolarons in the material. Conversely, for $\varepsilon_{Dedoped}$, the dedoped-state permittivity of PEDOT:PSS, $N = N_{Dedoped}$ and represents the number density of electrons (absence of polarons and bipolarons) in the material. These number densities can be expressed as a function of the total density of carriers participating in the electrochemical doping and undoping process, N_{Tot}, as follows:

$$
N_{Tot} = N_{Doped} + N_{Dedoped} = \alpha \cdot N_{Tot} + (1 - \alpha) \cdot N_{Tot}
$$
Equation S7

Where the doping level, $\alpha = \frac{N_{Doped}}{N_{Tot}}$, is defined as the ratio between the doped state and the total carrier density. From there, computing the PEDOT:PSS complex permittivity for intermediate doping levels ($0 < \alpha < 1$) comes down to a linear interpolation between the extreme permittivity $\varepsilon_{Doped}(\omega)$ and $\varepsilon_{Dedoped}(\omega)$ as per the following relation:

$$
\varepsilon_{PEDOT:PSS}(\alpha, \omega) = \alpha \cdot \varepsilon_{Doped}(\omega) + (1 - \alpha) \cdot \varepsilon_{Dedoped}(\omega)
$$
Equation S8

The resulting complex permittivities are presented in Figure S2-C and D.

1.3 Relationship between voltage biases and PEDOT:PSS complex permittivity

PEDOT doping levels can be adjusted by applying an external potential across the PEDOT:PSS material in an electrolyte (see Sub-section 1.1). The link between doping levels and voltage amplitudes was experimentally established by performing cyclic voltammetry while monitoring the relative variation of scattered light intensity (Z score) from PEDOT:PSS organic electro-scattering antennas (OCEANs, Figure S2-H). At positive voltages, the PEDOT is quasi-fully doped and Z score is minimum. At negative voltages, the PEDOT is quasi-fully dedoped and Z score is maximal. The linear voltage sweep across these two extreme voltage levels resulted in a sigmoidal relationship between Z score and the

applied voltage, which is reminiscent of the PEDOT:PSS doping level variations with voltage biases.(*43*) Assuming that the optical signal is only modulated by a change in PEDOT:PSS doping levels, fitting it with a sigmoid function enables the expression of the relationship between doping levels and voltage amplitudes as follows:

$$\alpha(V) = \frac{1}{1 + e^{-\frac{(V - V_0)}{\beta}}} \quad \text{Equation S9}$$

Where $V_0 = -445\ mV$ is the sigmoid centering voltage where $\alpha = 0.5$, and $\beta = 135\ mV$ is a fitting parameter that defines the steepness of the transition.

The PEDOT:PSS complex permittivity can therefore be expressed as a function of the applied voltage across the PEDOT:PSS-phosphate-buffered saline (PBS) interface by the following expression:

$$\varepsilon_{PEDOT:PSS}(V, \omega) = \alpha(V) \cdot \varepsilon_{Doped}(\omega) + (1 - \alpha(V)) \cdot \varepsilon_{Dedoped}(\omega) \quad \text{Equation S10}$$

Figure S2-E and F show the real and imaginary parts of the PEDOT:PSS permittivity under different voltage bias conditions. The variation of the real part of the permittivity with respect to wavelength for different bias levels is shown in Figure S2-G. At negative bias voltages and large wavelengths, the real permittivity of PEDOT:PSS exhibits maximum sensitivity to voltage.

2 Nanofabrication of OCEANs

The complete process flow developed to manufacture and interface OCEANs, as well as the method used to systematically characterize the OCEAN geometries following each fabrication batch, are presented and discussed in detail in this section.

2.1 Extended nanofabrication of OCEANs

Figure S3-A illustrates the complete nanofabrication process developed to manufacture OCEANs. The process started with a 170 µm-thick indium tin oxide (ITO) -coated glass substrate. First, the 70 nm-thick ITO layer was patterned by chlorine-based reactive ion etching to define conductive traces and pads, eventually enabling electrical access to OCEANs. These ITO structures were subsequently passivated with a 50 nm-thick layer of silicon nitride (SiN_x) deposited by plasma-enhanced chemical vapor deposition. A 3 µm-thick layer of SU8 was patterned with 80 µm square openings by photolithography to maximize the electrical trace stray impedances while permitting direct access to the ITO-SiN_x pads for OCEAN patterning. Focused-ion beam (FIB) lithography was used to pattern arrays of 250 nm in diameter nanoholes in the exposed SiN_x layer. Electrodeposition of PEDOT:PSS was then performed to locally grow OCEANs from each cavity and form OCEAN arrays.

The OCEAN chip was electrically interfaced with a custom-designed printed circuit board (PCB) – before PEDOT:PSS electrodeposition – by dispensing conductive epoxy on each PCB interfacing pad and employing a die bonder to position the chip on the PCB precisely (Figure S3-B). The PCB comprised four conductive layers. The two outer layers formed ground planes that shielded the two inner layers patterned with electrical traces connecting the PCB standard connectors to the different electrical sections of the chip. Each chip comprised 25 arrays of OCEANs – each composed of 16 x 16 OCEANs – electrically interfaced by 12 conductive traces (2 arrays per conductive trace for the first 11 traces and 3 arrays for the last one). Any electrical instrument could independently address each trace using the 12-position DIP switch connected to a standard U.FL connector marked WE for working electrode. The two other U.FL connectors designated as CE and REF for counter and reference electrodes, respectively, were directly connected to standard pin connectors where platinum and silver/silver chloride (Ag/AgCl) reference electrodes could easily be mounted and positioned to contact the bath. The custom-designed PCB permitted robust and low-noise interfacing of OCEANs with a three-electrode potentiostat and strongly facilitated their fabrication and characterization. The Omnetics connector on the right side of the PCB will be used in subsequent work to interface an array of microelectrodes seamlessly integrated into OCEANs and used as control. The U.FL connector labeled STIM for stimulation will be used to interface an integrated stimulation dipole, potentially useful to pace the culture.

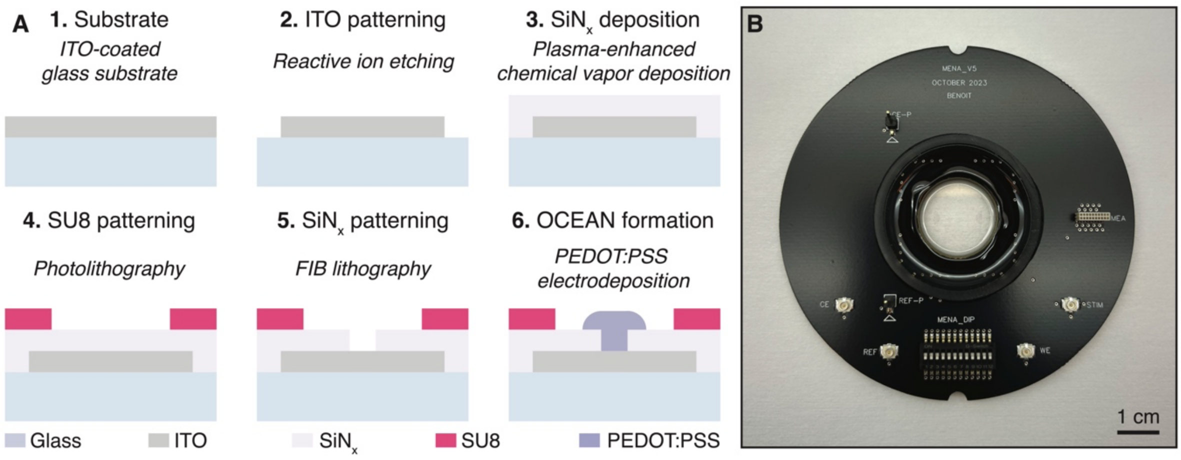

Figure S3: Nanofabrication and interfacing of OCEANs. A) Nanofabrication process flow developed to manufacture OCEANs. B) Optical image of the device following electrical and mechanical interfacing with a custom-designed PCB. A glass ring is mounted on top to facilitate experiments in liquid.

2.2 Geometrical characterization of OCEANs

To account for potential variability in the process, the PEDOT:PSS electrodeposition step was systematically monitored under an optical microscope in real-time. At the end of the process, a custom algorithm was used to estimate the diameter of the freshly developed OCEANs and their variability. Figure S4 presents the algorithm's outcome for the three electrodeposition times (t_{ED}) studied in this work. Assuming an isotropic electrodeposition process, the heights of the antenna cap (H_{OCEAN}) can be estimated from the cap and stem diameter ($\emptyset_{Cap}$ and $\emptyset_{Stem}$, respectively) using the following equation.

$$H_{OCEAN} = \frac{\emptyset_{Cap} - \emptyset_{Stem}}{2} \qquad \text{Equation S11}$$

Consequently, the height of the small, intermediate, and large antennas (*i.e.,* $\emptyset_{Cap}$ = 0.7 µm, 1.4 µm, and 1.8 µm) was estimated to be 225 nm, 575 nm, and 775 nm, respectively ($\emptyset_{Stem}$ = 250 nm).

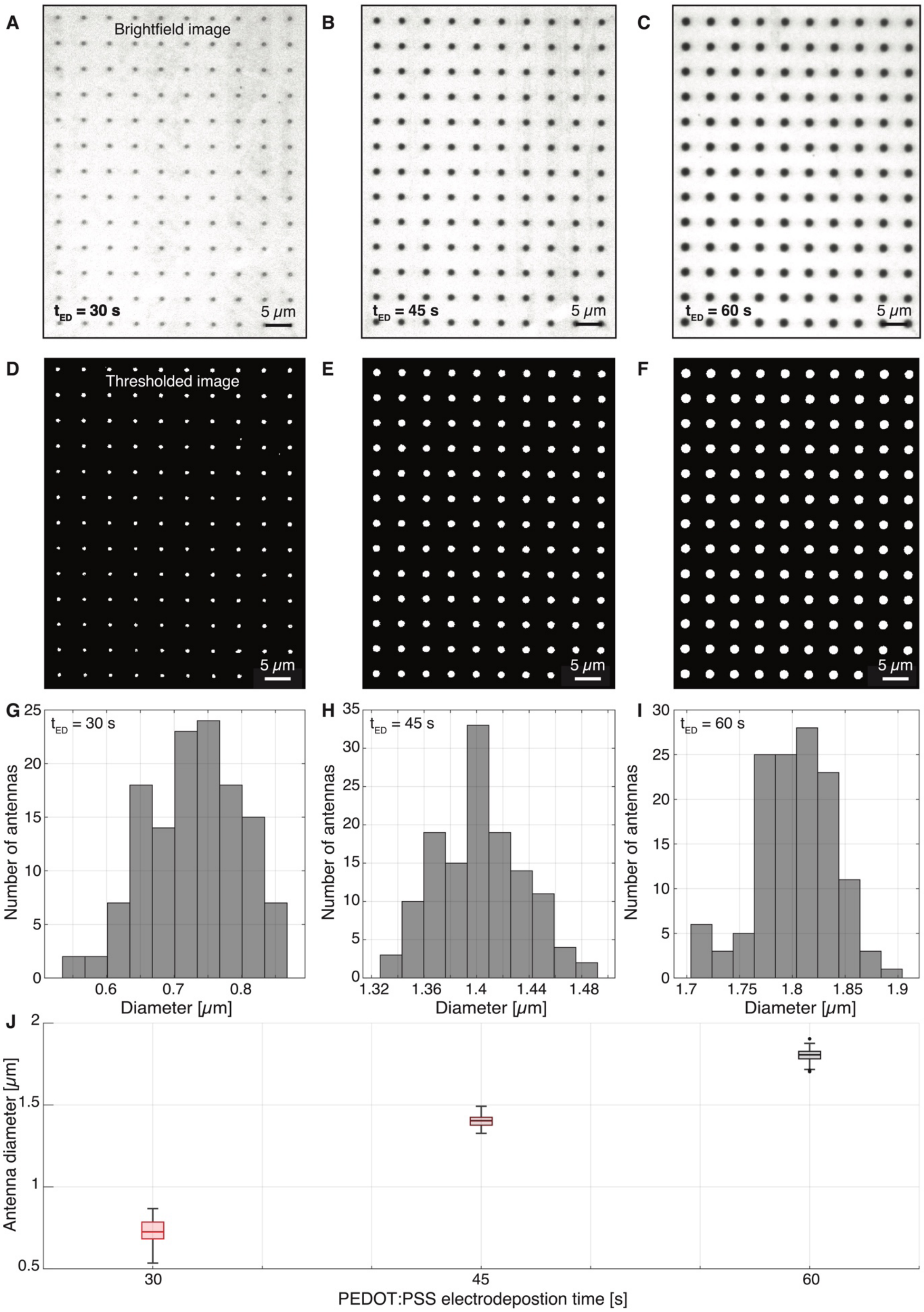

Figure S4: OCEAN diameter analysis by optical microscopy. Optical microscopy image of an array of OCEANs following A) 30 s, B) 45 s, and C) 60 s of PEDOT:PSS electrodeposition. The images were made with a 60x water dipping objective. D) to F) Corresponding thresholded images established from

A) to C) and used to determine the cap diameters and variability. G) to I) Distributions of cap diameters extracted from thresholded images D) to F) for the three different electrodeposition times tested. J) Box chart summarizing the relationship between PEDOT:PSS electrodeposition time and cap diameter (N = 130). These OCEANs were grown through SiN_x openings of 250 nm in diameter.

3 Electrochemical characterization of OCEANs

The electrochemical properties of OCEANs were systematically characterized in PBS. The outcome of this procedure is presented in detail in the following section. Experimental data acquired by electrochemical impedance spectroscopy (EIS) are first introduced and discussed, and then followed by the implementation of a simple equivalent electrical circuit describing the PEDOT:PSS OCEAN-electrolyte interface.

3.1 Electrochemical impedance spectroscopy on OCEANs

Figure S5 presents the EIS experimental data acquired for OCEAN arrays composed of OCEANs with diameters ranging from 0.7 µm to 1.8 µm and voltage biases between 0 V and -0.6 V. At fixed bias, the OCEAN-electrolyte interfaces behave similarly to the conventional microelectrode-electrolyte interfaces, suggesting a double layer constant phase element in serial with a spreading resistance, and in parallel with a stray capacitance as an electrical equivalent circuit.(*60*) However, an additional resistance, whose value is modulated by the bias voltage amplitude, is necessary to explain the EIS plot's dependence on voltage (Figure S6-A). This resistance, $R_{PEDOT:PSS}$, represents the electrical resistance of the PEDOT strands and becomes particularly significant when PEDOT:PSS becomes dedoped.

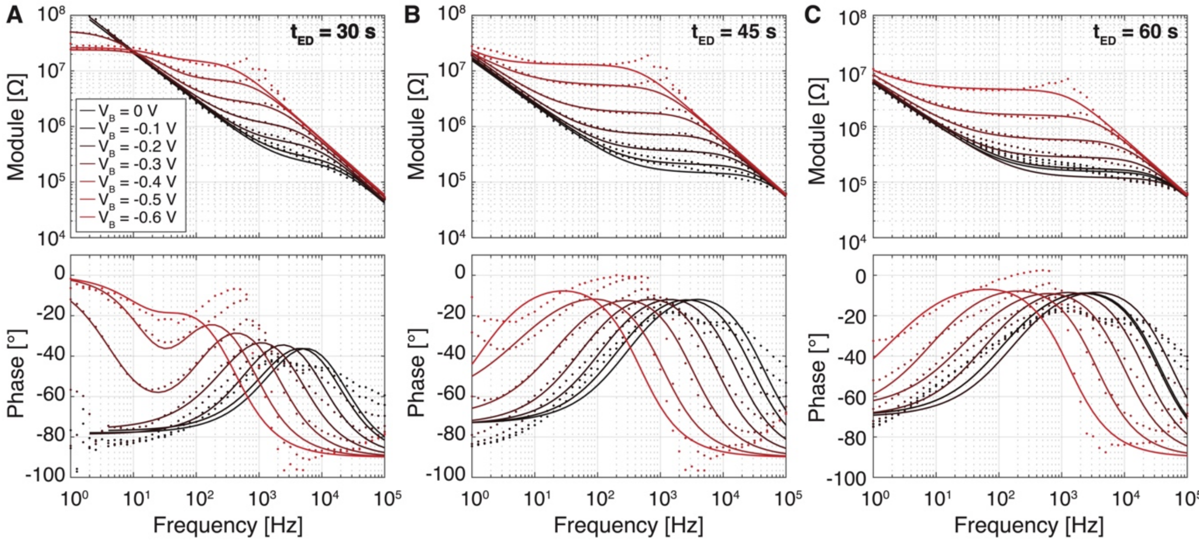

Figure S5: Electrochemical impedance spectroscopy on OCEANs. Impedance module (top) and phase (bottom) of PEDOT:PSS OCEAN arrays (2 x 16 x 16 = 512 OCEANs), versus frequency, for different voltage biases in PBS and cap diameters ranging from A) 0.7 µm (t_{ED} = 30s), B) 1.4 µm (t_{ED} = 45s), to C) 1.8 µm (t_{ED} = 60s). Dots and solid curves represent the experimental data and theoretical fit based on the electrical equivalent circuit shown in Figure S6-A, respectively.

3.2 Modeling of the OCEAN-electrolyte interface

Figure S6-A displays the electrical equivalent circuit implemented to fit the EIS experimental data presented in Figure S5. It comprises a constant phase element $CPE_{PEDOT:PSS}$ representing the transfer of charge at the PEDOT:PSS-electrolyte interface (of impedance $Z_{CPE} = \frac{1}{Q_0(j\omega)^n}$, where Q_0 is a measure of Z_{CPE} magnitude, n is a constant between 0 and 1, and ω is the pulsation), a spreading resistance R_s, a stray capacitance C_{Stray}, and a voltage-dependent resistance $R_{PEDOT:PSS}$ accounting for the loss of conductivity in PEDOT during dedoping.(*37*) Figure S6-B to E summarize the numerical value of each component included in the electrical equivalent circuit and how they are modulated by the OCEAN geometry and operating voltage. The computed respective time constants $\tau = (R_{PEDOT:PSS} + R_s) \cdot C_{PEDOT:PSS}$ are presented in Figure S6-F. The interface was assumed to be purely capacitive for simplification purposes, and consequently, $C_{PEDOT:PSS}$ was used instead of $CPE_{PEDOT:PSS}$ ($Z_{CPEDOT:PSS} = Z_{CPE}\ for\ n = 1$). At negative biases, the OCEAN kinetics were anticipated to become slower due to the increased $R_{PEDOT:PSS}$. Additionally, larger OCEANs were expected to show slower time constants due to larger capacitances. Nevertheless, increases in $R_{PEDOT:PSS} + R_S$ partially compensated for the increase in $C_{PEDOT:PSS}$ in the case of the largest OCEANs (t_{ED} = 60 s), and, therefore, their time constants remained comparable to OCEANs of intermediate size.

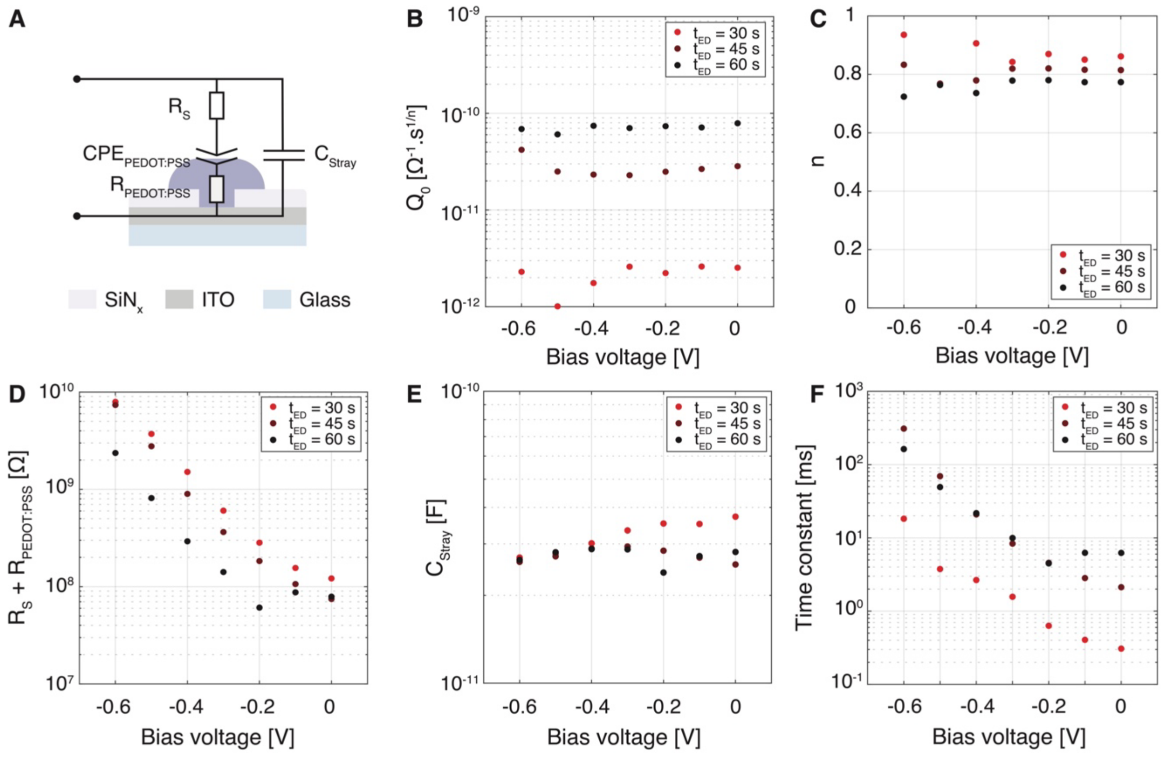

Figure S6: Electrical equivalent circuit of single OCEAN-electrolyte interfaces. A) Schematic representation of the electrical equivalent circuit of an OCEAN-electrolyte interface. Plots showing B) Q_0, C) n, D) $R_S + R_{PEDOT:PSS}$, and E) C_{Stray} with respect to voltage bias amplitudes for small (t_{ED} = 30 s, diameter 0.7 µm), intermediate (t_{ED} = 45 s, diameter 1.4 µm), and large (t_{ED} = 60 s, diameter 1.8 µm)

cap diameters. Numerical values extracted from the EIS experimental dataset shown in Figure S5. F) Time constants computed by multiplying $R_{PEDOT:PSS} + R_s$ with $C_{PEDOT:PSS}$ for each condition investigated.

4 Electro-optic characterization of OCEANs

This section provides a comprehensive description of the experimental setup used to characterize the electro-optic characteristics of OCEANs and presents the complete dataset collected during this process.

4.1 Relative irradiance spectra of OCEAN arrays for different voltage biases

Relative irradiance spectra of OCEAN arrays (50 x 50 OCEAN per array, ~1.8 µm cap diameter) were acquired in PBS while applying different voltage biases between the ITO and the bath. The OCEAN arrays were illuminated by a broadband light source through the diascopic dark-field condenser of an inverted microscope. The light scattered by the array was collected by a 40x objective and analyzed by an optical spectrometer connected to the output port of the microscope via an optical fiber. The acquired spectra were normalized by the light source spectrum to compensate for its non-uniformity. Voltage biases were applied across the OCEANs using a potentiostat in a three-electrode configuration, where the ITO acted as the working electrode, an Ag/AgCl reference electrode as the reference electrode, and a platinum wire as the counter electrode. The resulting spectra are presented in Figure S7. At negative voltages, the PEDOT composing the OCEANs becomes dedoped, which increases the real part of its permittivity (Figure S2-E). As a result, the OCEANs are more effective at scattering light.

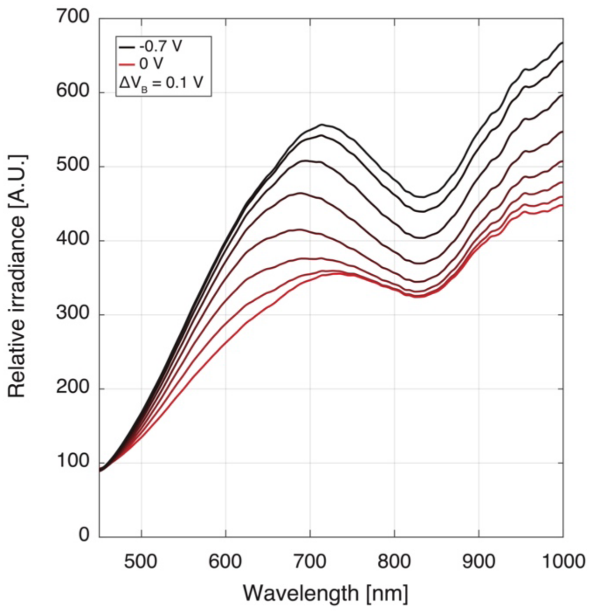

Figure S7: OCEAN irradiance spectra. Relative irradiance spectra of PEDOT:PSS OCEAN arrays for different bias voltages in PBS.

4.2 Total internal reflection darkfield microscopy

A custom total internal reflection darkfield microscopy setup was developed to confine the incident illumination to the plane defined by the array of OCEANs and collect only the light they scattered. The optical setup comprises an upright microscope body coupled to a complementary metal-oxide

semiconductor (CMOS) camera, a 60 x water dipping objective, a multiwavelength light source to image the device using reflected brightfield microscopy, and a custom-designed holder mounted on a motorized XY stage (Figure S8-A and C). To confine the illumination to the plane defined by the OCEANs, a prism-based total internal reflection illumination module was integrated into the setup (Figure S8-B). The output of a 637 nm pig-tailed laser diode was collimated, mounted at an angle of 30° with respect to the horizontal plane, and aligned with an N-BK7 prism to illuminate OCEANs above the critical angle and achieve total internal reflection. Immersion oil was placed between the prism and the sample to ensure a continuous high-refractive index medium. Under these conditions, an evanescent wave propagated vertically towards the electrolyte with an amplitude decreasing exponentially in the direction of propagation, thus confining the excitation light to the first hundreds of nanometers following the SiN_x-electrolyte interface. Such illumination will permit the minimization of background scattering from cells and, therefore, will play a central role in maintaining a low limit of detection when translating to biological experiments. Furthermore, because the laser is collimated, it enables the imaging of entire arrays of OCEANs with submicrometer spatial resolution, facilitating data collections during characterization workflows and, potentially, enabling wireless electrophysiological studies in cell networks with subcellular resolution. Note that the incident illumination should come from the direction opposite to the microscope objective to collect the forward scattering from the OCEANs, whose intensity is brighter than the backward scattering. A three-electrode potentiostat was used to apply voltage stimuli across OCEANs using an external platinum wire and an Ag/AgCl reference electrode as counter and reference electrodes, respectively.

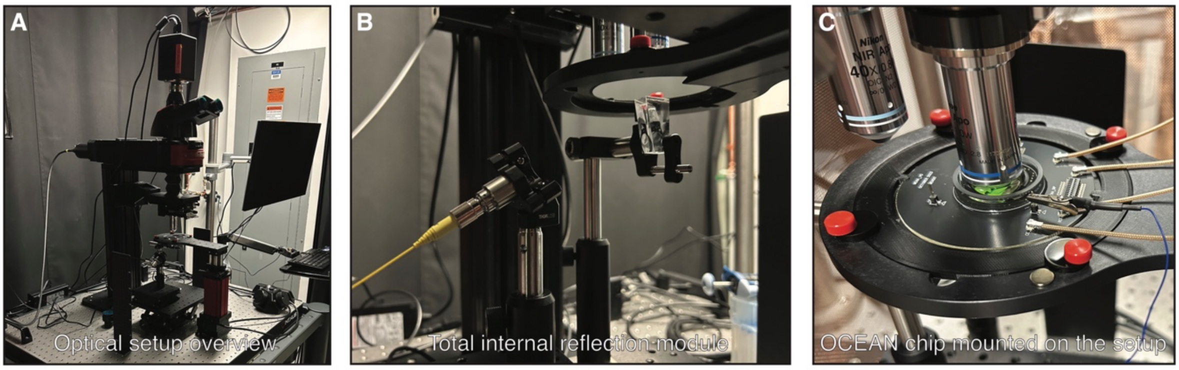

Figure S8: Total internal reflection dark-field microscopy. A) Optical image showing the experimental setup developed to characterize the electro-optic characteristics of OCEANs. B) Picture of the prism-based total internal reflection illumination module. C) Image of an OCEAN chip mounted on the experimental setup and electrically connected to a potentiostat. The diascopic green light is used to image the device using reflected brightfield microscopy.

4.3 Optical and electrical cyclic voltammetry with OCEANs

Figure S9-A to C show optical and electrical voltammograms measured from OCEANs of different dimensions. The two different cycles composing each optical and electrical plot show, overall, similar

behaviors. However, electrical traces displayed redox currents at voltages around -0.1 V and 0.1 V. Interestingly, their amplitude decreased with cycles, and their contribution to the optical signal variation seemed minimal, especially for larger OCEANs. Figure S9-D to F show total internal reflection dark-field microscopy images of OCEAN arrays of different dimensions under a voltage bias of -0.8V vs Ag/AgCl.

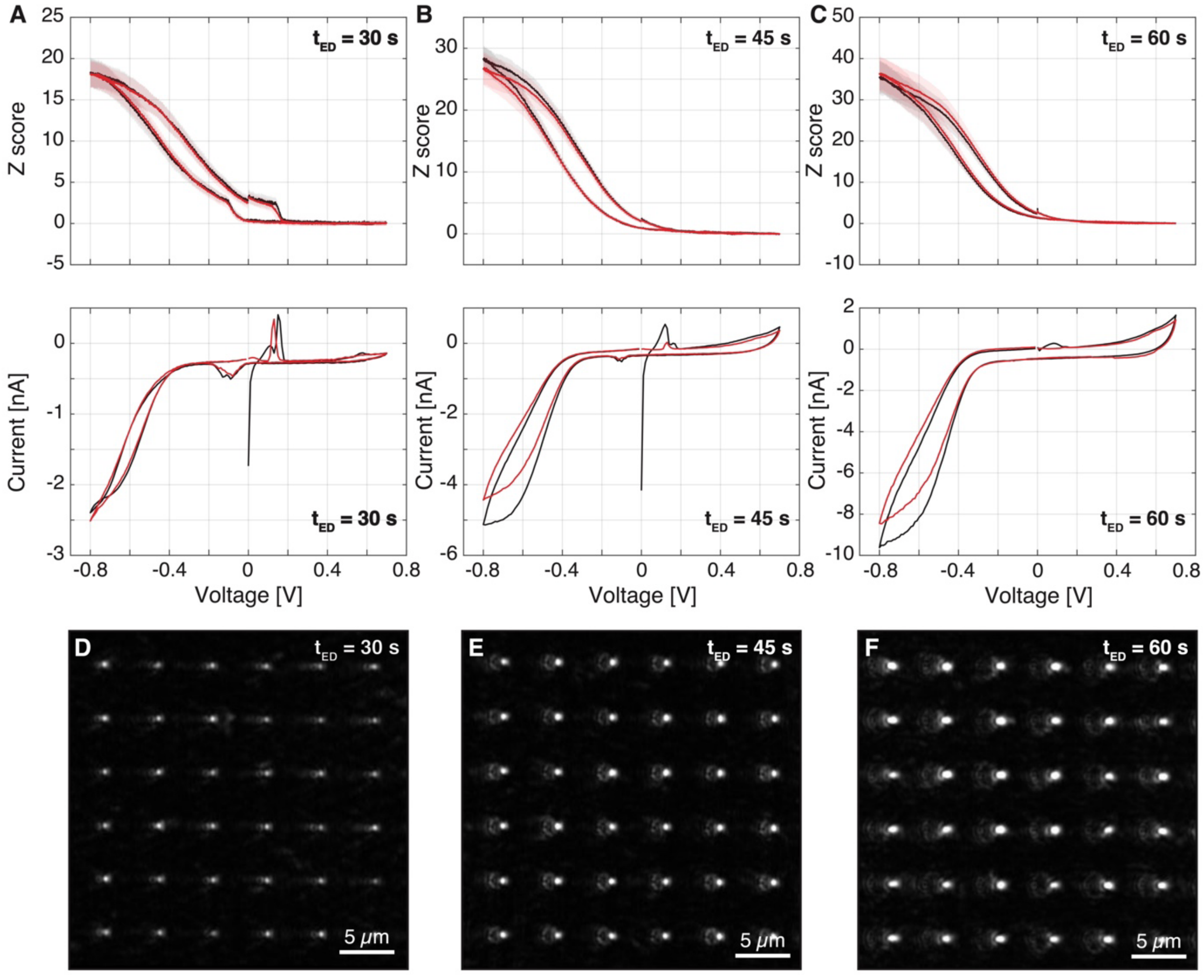

Figure S9: Electrical and optical cyclic voltammetry with OCEANs. Optical (top) and electrical (bottom) voltammograms measured from OCEANs of A) small (t_{ED} = 30 s, diameter 0.7 µm), B) intermediate (t_{ED} = 45 s, diameter 1.4 µm), and C) large (t_{ED} = 60 s, diameter 1.8 µm) dimensions. The optical traces were constructed by averaging the individual voltammogram of each OCEAN composing the array (N = 16, mean ± standard deviation). The electrical voltammogram displays the current going through the entire array (512 OCEANs). The black curve represents the first cycle, while the red represents the second one. Potentials are swept with respect to an Ag/AgCl reference electrode. Total internal reflection dark-field microscopy images of D) small, E) intermediate, and F) large OCEAN arrays under a voltage bias of -0.8V vs Ag/AgCl.

4.4 Electro-optic responses of OCEAN to voltage pulses

The influence of cap diameters and operating biases on OCEAN electro-optic performance was systematically characterized by applying voltage pulses of amplitudes ranging from -100 mV to 100

mV across OCEANs in PBS while monitoring their optical Z score individually. Figure S10 to S12 present the overall resulting dataset.

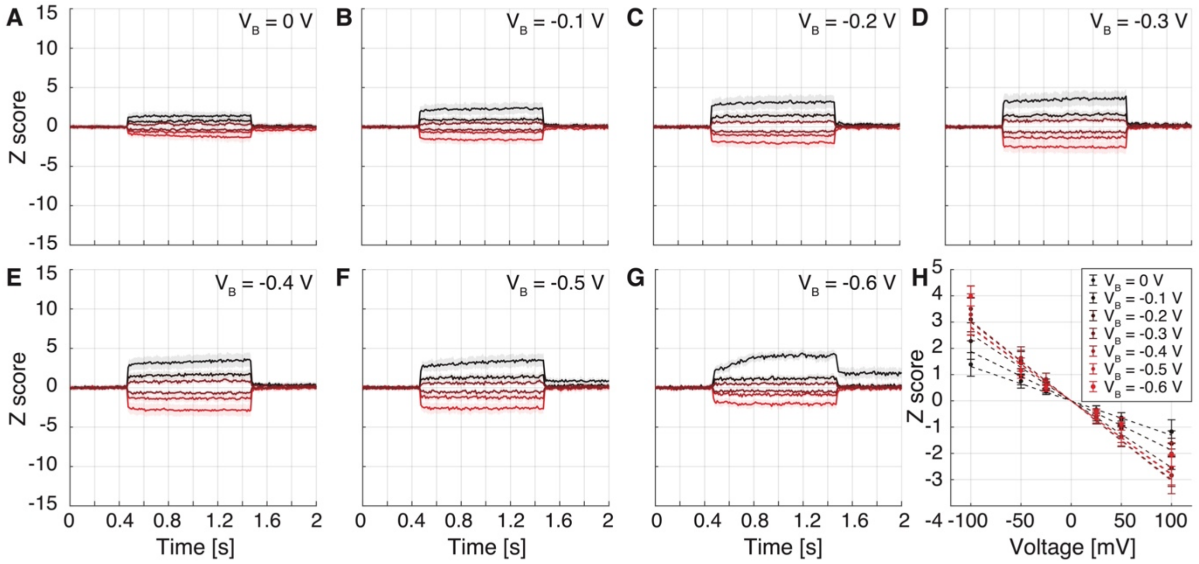

Figure S10: Electro-optic modulation of small OCEANS. Electro-optic modulation of single OCEANs (t_{ED} = 30 s, diameter 0.7 µm) in response to voltage pulses with amplitude ranging from -100 mV (black), -50 mV, -25 mV, 25 mV, 50 mV, to 100 mV (red) in PBS. Operating biases are swept from A) 0V, B) -0.1 V, C) -0.2 V, D) -0.3 V, E) -0.4 V, F) -0.5 V, to G) -0.6 V with respect to Ag/AgCl reference electrode. H) Optical Z score with respect to voltage pulse amplitude for the different operating biases tested. (N=16, mean ± standard deviation).

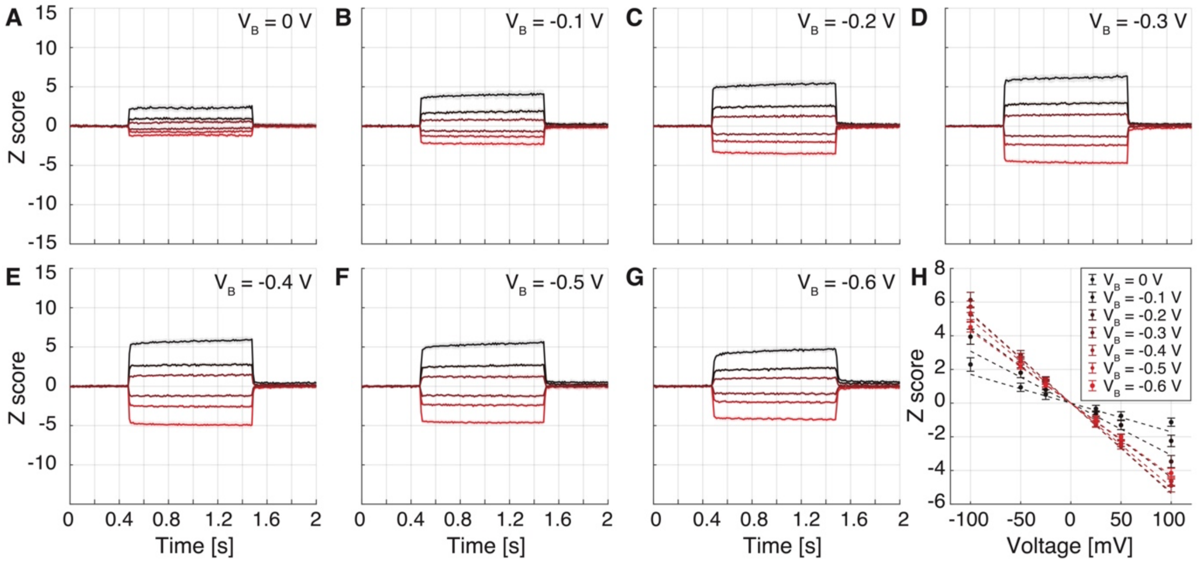

Figure S11: Electro-optic modulation of intermediate OCEANS. Electro-optic modulation of single OCEANs (t_{ED} = 45 s, diameter 1.4 µm) in response to voltage pulses with amplitude ranging from -100 mV (black), -50 mV, -25 mV, 25 mV, 50 mV, to 100 mV (red) in PBS. Operating biases are swept from A) 0V, B) -0.1 V, C) -0.2 V, D) -0.3 V, E) -0.4 V, F) -0.5 V, to G) -0.6 V with respect to Ag/AgCl reference electrode. H) Optical Z score with respect to voltage pulse amplitude for the different operating biases tested. (N=16, mean ± standard deviation).

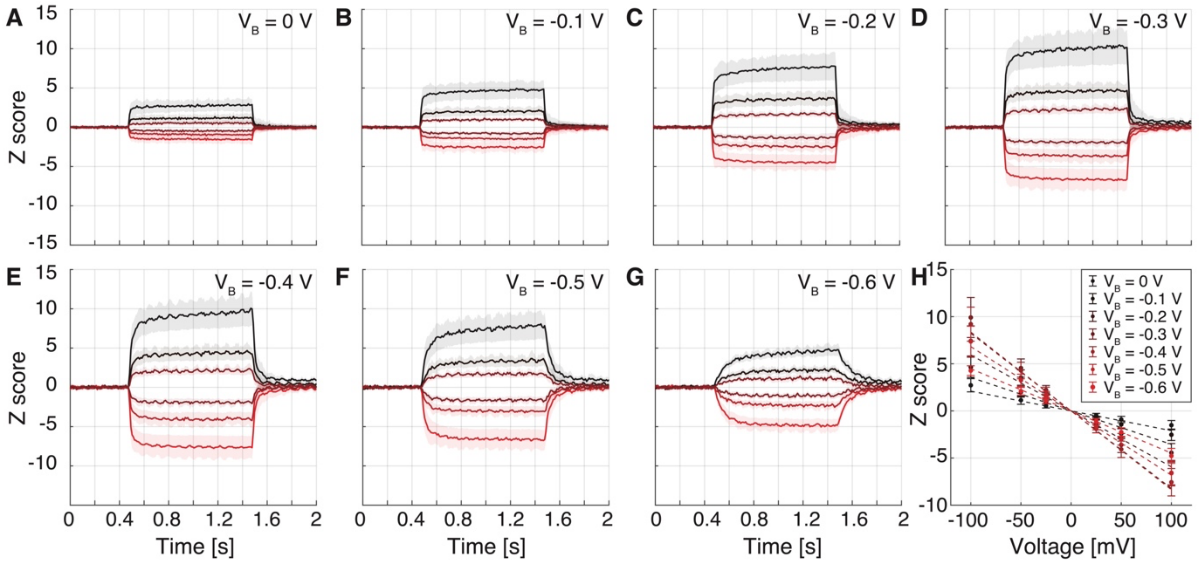

Figure S12: Electro-optic modulation of large OCEANS. Electro-optic modulation of single OCEANs (t_{ED} = 60 s, diameter 1.8 µm) in response to voltage pulses with amplitude ranging from -100 mV (black), -50 mV, -25 mV, 25 mV, 50 mV, to 100 mV (red) in PBS. Operating biases are swept from A) 0V, B) -0.1 V, C) -0.2 V, D) -0.3 V, E) -0.4 V, F) -0.5 V, to G) -0.6 V with respect to Ag/AgCl reference electrode. H) Optical Z score with respect to voltage pulse amplitude for the different operating biases tested. (N=16, mean ± standard deviation).

4.5 Dynamic characteristics of OCEANs

The time constants of single OCEANs were experimentally determined for different cap diameters and operating biases. 200 ms-long and 100 mV amplitude voltage pulses were applied across the OCEANs in PBS while monitoring their optical signal at 2’000 frames per second. The optical traces from each OCEAN were fitted with an exponential to estimate their time constant. Figure S13-A to C present the average experimental and fitted optical traces used to study the influence of cap geometries and operating biases on the dynamic characteristics of OCEANs. Main manuscript Figure 4-H summarizes the experimentally measured time constants for the different conditions tested.

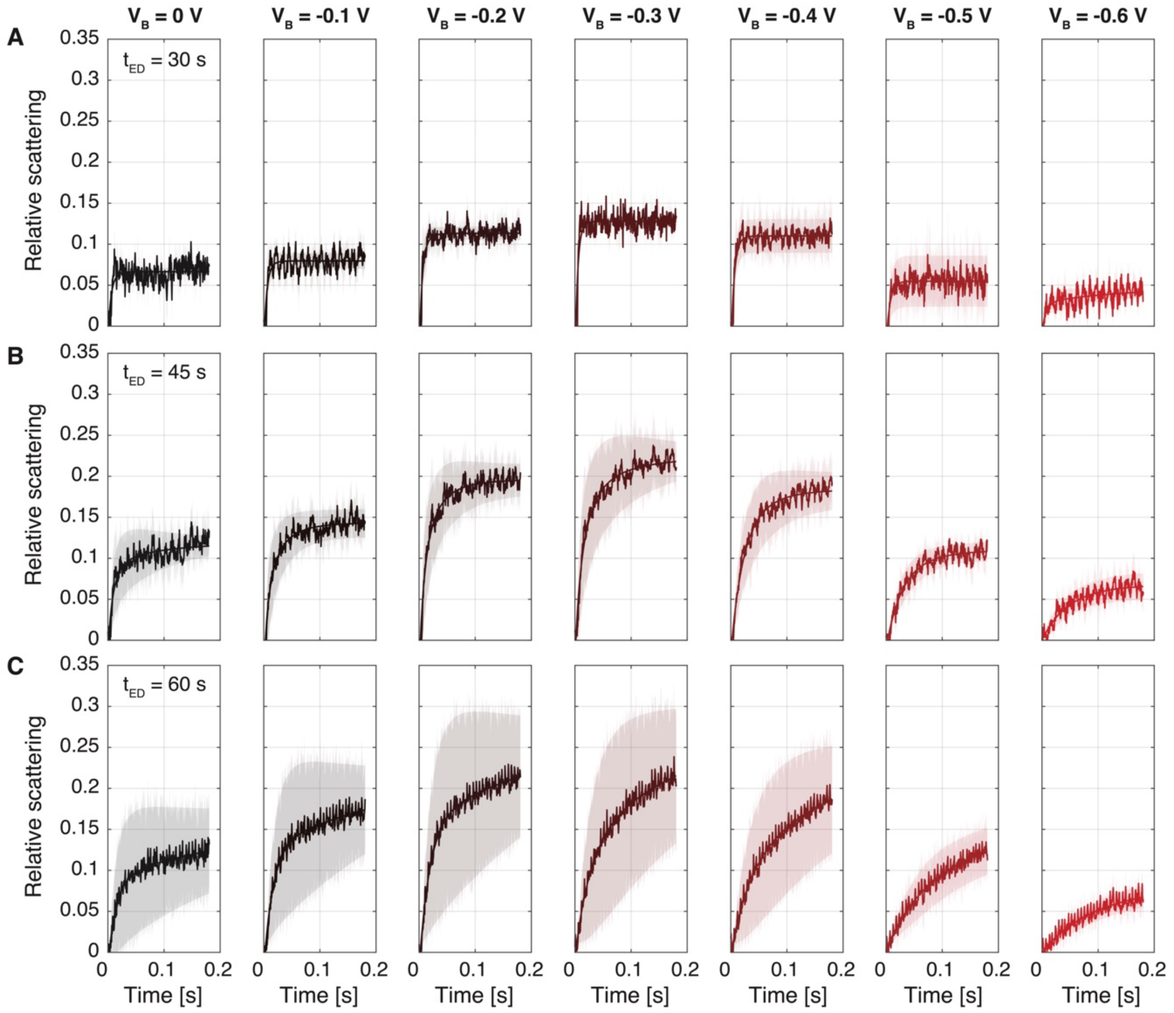

Figure S13: Dynamic characteristics of OCEANs. Optical traces and exponential fits collected from single OCEANs with diameters ranging from A) 0.7 µm (t_{ED} = 30 s), B) 1.4 µm (t_{ED} = 45 s), to C) 1.8 µm (t_{ED} = 60 s) for operating biases between 0 V and -0.6 V with respect to Ag/AgCl reference electrode (N=6, mean ± standard deviation).

4.6 Long-term stability of OCEANs

The long-term stability of OCEANs was investigated by applying a 1 Hz and 100 mV in amplitude voltage square wave with respect to an operating bias of -0.3 V for 10 hours. The optical responses of single OCEANs were intermittently monitored throughout the experiment and are presented in Figure S14. Interestingly, the average Z score only decreased from 6 to 5 following 36'000 electrochemical modulation cycles, demonstrating the remarkable long-term stability of most OCEANs (main manuscript Figure 4-G). Nevertheless, both the standard deviations and the number of outliers also increased throughout the duration of the experiment, suggesting that some OCEANs showed deteriorated electro-optic properties. No morphological differences were observed between the OCEANs showing long-term stability versus the other ones. This heterogeneity could originate from a deterioration of the ITO-PEDOT:PSS interface with time, potentially through delamination, hindering electrochemical doping and dedoping in some OCEANs. Alternatively, variability in the structural re-

organization of PEDOT:PSS structure in response to cyclic stimulation could also explain the deteriorated electro-optic properties. For instance, the repeated injection of solvated cations in the PEDOT:PSS structure could wash away excess of the hydrophilic poly(sodium 4-styrene sulfonate) and affect the electro-optic properties of the OCEAN. In such a case, post-treatment with strong sulfuric acid could be investigated to remove the excess of poly(sodium 4-styrene sulfonate) and re-organize PEDOT:PSS before experiments.(*61*) The slower OCEAN kinetics observed after hours of stimulation suggests a steady dedoping of PEDOT:PSS, which could potentially be explained by similar phenomena. Nevertheless, further investigations should be performed to accurately identify the underlying mechanisms limiting the long-term stability of some OCEANs.

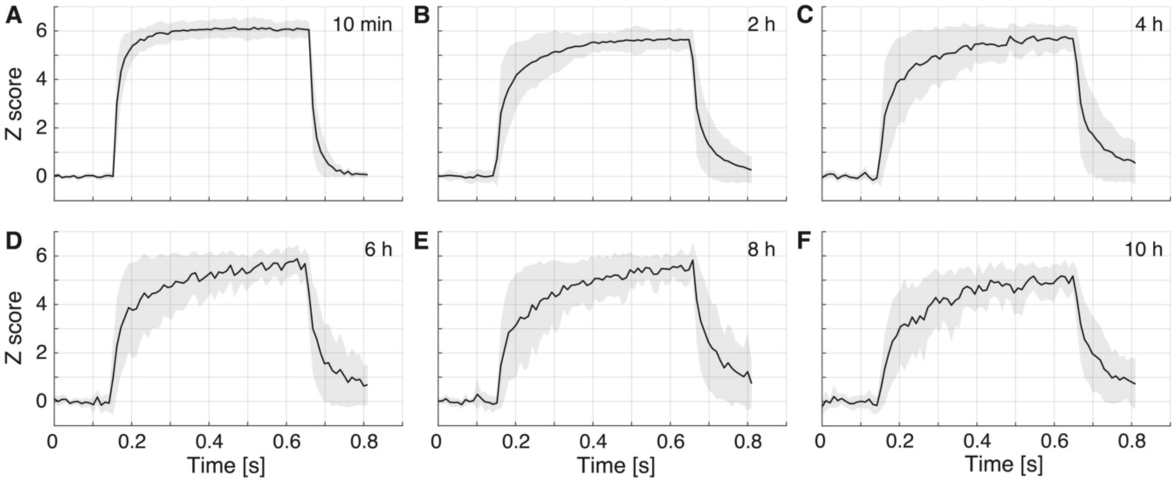

Figure S14: Long-term stability of OCEANs. A) to F) Electro-optic modulation of single OCEANs (1.4 µm in diameter) following 10 min and up to 10 hours of electrical stimulation with 500 ms-long and -100 mV voltage pulses at 1 Hz (V_B = -0.3 V, N=16, mean ± standard deviation).

4.7 Performance summary and comparison to state-of-the-art voltage-to-light transducers

Table S2 summarizes OCEAN electro-optic characteristics with respect to electrodeposition time and bias voltage.

Table S2: OCEAN electro-optic characteristics. Summary of OCEAN experimental electro-optic characteristics with respect to voltage bias and antenna diameter (mean ± standard deviation)

Voltage bias [V]	Sensitivity [mV^{-1}]			Noise			SNR			Limit of detection [mV]			Time constant [ms]		
**t_{ED} [s]**	**30**	**45**	**60**	**30**	**45**	**60**	**30**	**45**	**60**	**30**	**45**	**60**	**30**	**45**	**60**
**0**	-0.013 ± 0.004	-0.017 ± 0.003	-0.021 ± 0.006	0.234 ± 0.064	0.130 ± 0.039	0.193 ± 0.065	6.19 ± 2.51	18.46 ± 4.13	14.90 ± 3.32	22.58 ± 21.18	7.67 ± 2.11	9.10 ± 1.62	6.1 ± 1.8	29.9 ± 42.7	175.0 ± 260.3
**-0.1**	-0.019 ± 0.006	-0.031 ± 0.004	-0.036 ± 0.008	0.237 ± 0.085	0.123 ± 0.017	0.174 ± 0.065	10.58 ± 4.36	32.52 ± 5.10	28.08 ± 5.16	15.35 ± 13.61	4.06 ± 0.84	4.80 ± 0.85	5.6 ± 1.9	19.3 ± 15.7	141.5 ± 171.2
**-0.2**	-0.025 ± 0.007	-0.044 ± 0.003	-0.059 ± 0.013	0.246 ± 0.085	0.127 ± 0.021	0.191 ± 0.061	14.08 ± 5.92	42.40 ± 7.33	40.30 ± 7.09	10.76 ± 6.25	2.90 ± 0.51	3.20 ± 0.56	6.1 ± 3.0	21.8 ± 19.2	228.0 ± 346.5
**-0.3**	-0.03 ± 0.008	-0.054 ± 0.004	-0.082 ± 0.017	0.246 ± 0.082	0.132 ± 0.026	0.196 ± 0.065	16.08 ± 6.82	48.18 ± 10.93	53.47 ± 11.05	9.23 ± 5.72	2.47 ± 0.49	2.34 ± 0.51	4.8 ± 0.6	34.7 ± 39.1	235.4 ± 284.2
**-0.4**	-0.031 ± 0.007	-0.053 ± 0.003	-0.084 ± 0.016	0.242 ± 0.078	0.137 ± 0.021	0.192 ± 0.071	14.94 ± 5.66	42.52 ± 5.75	51.66 ± 11.76	8.67 ± 4.34	2.59 ± 0.42	2.23 ± 0.50	6.0 ± 1.1	32.7 ± 20.4	233.9 ± 299.4
**-0.5**	-0.028 ± 0.006	-0.050 ± 0.003	-0.068 ± 0.014	0.246 ± 0.094	0.146 ± 0.030	0.217 ± 0.074	15.13 ± 6.47	38.00 ± 8.54	35.88 ± 6.67	9.48 ± 5.25	2.96 ± 0.67	3.10 ± 0.58	7.0 ± 0.8	36.6 ± 19.4	120.7 ± 54.0
**-0.6**	-0.028 ± 0.003	-0.043 ± 0.003	-0.045 ± 0.006	0.249 ± 0.082	0.150 ± 0.037	0.213 ± 0.085	17.91 ± 6.58	31.82 ± 8.23	23.1563 ± 9.2596	9.12 ± 3.44	3.49 ± 0.80	4.66 ± 1.49	81.4 ± 99.6	50.5 ± 16.2	119.1 ± 70.9

Table S3 summarizes the electro-optic performance characteristics of OCEANs compared to state-of-the-art transducers. To ensure a meaningful comparison, we specifically focused on wireless technologies that operate under conditions analogous to those of OCEANs, allowing us to extract and directly compare relevant performance characteristics.

Table S3: OCEAN performance compared to the state-of-the-art. Performance comparison between OCEANs and state-of-the-art voltage-to-light transducers

Technology	Number of recording sites	Spatial resolution	Limit of detection	Time constant
Ecore(*26*)	Single	Probe diameter: 25 µm	5 µV	0.2 ms
Electroplasmonic nanoantennas(*29*)	Single	Probe diameter: 100's µm♦	~10's mV*	0.191 ms
**This work**	Up to 12'000	Probe diameter: 1.4 µm<br>Pitch: 5 µm	2.5 mV	26.8 ms

♦Estimated from the 20x objective field of view diameter used in Figure 4 of reference(*29*)
*Estimated from Figure 4-B of reference(*29*)

5 Modeling intracellular cardiomyocyte action potential recording with OCEANs

As a case study, we investigated the feasibility of using OCEANs to record cardiomyocyte action potentials wirelessly following intracellular access by electroporation. The protruding geometry of the OCEAN, together with its ~1 µm dimension, promotes an enhanced coupling at the cell-OCEAN interface and, therefore, a large seal resistance.(*45*) The electrically insulated substrate also significantly contributes to minimizing the current leaks. A large seal resistance is essential to minimize the electrophysiological signal attenuation at the cell-OCEAN interface and guarantees recordings of quality.(*62*) It also plays a central role in enabling intracellular access to the OCEAN following electroporation, minimizing the required electroporation voltage amplitude, and confining pore formations to the junctional cell membrane.(*63*) Under these conditions, the cell transmembrane potential is anticipated to be efficiently transferred across the PEDOT:PSS structure with minimal attenuation and to modulate its scattering properties.

5.1 Modeling the cardiomyocyte-OCEAN interface

An electrical equivalent circuit of the cell-OCEAN interface was developed to predict how much of the electrophysiological signal can be transferred across the OCEAN under two different electrical configurations: pseudo-current clamp and voltage clamp. A pseudo-current clamp configuration represents the electrical arrangement used in multi-electrode arrays (MEAs), where the ideally infinite input impedance of the amplifier separates the electrode from the ground. However, under this condition, no voltage bias can be applied across the OCEAN, therefore minimizing their electro-optic sensitivity. On the other hand, a pseudo-voltage clamp configuration would permit the application of an operating bias voltage across OCEANs. Nevertheless, the bias voltage hypothetical clamping effects on the transmembrane potentials of cells should be investigated. In this section, we analyzed these two different electrical arrangements to better understand their respective implications on OCEAN recording capabilities and their potential adverse effects on cell electrophysiology. A Luo Rudy model was used to simulate the electrogenic characteristics of a ventricular cardiomyocyte.(*64*)

Figure S15-A shows a schematic representation of the electrical equivalent circuit developed to study the feasibility of electrophysiological recordings using OCEANs in a pseudo-current clamp configuration. Due to the infinite input impedance of the current source connected between the ITO and the bath, the current passing through the PEDOT:PSS structure is purely dictated by the current source. When a small stimulation current pulse I_{Stim} is applied (Figure S15-B, left), the transmembrane potential V_m becomes slightly depolarized, while $V_{PEDOT:PSS}$ only reflects the charge of the capacitance $C_{PEDOT:PSS}$ in response to I_{Stim}, independently from V_m. At the end of the current pulse, V_m returns to its resting value, and $C_{PEDOT:PSS}$ discharges through the equivalent circuit; however, with a slower kinetic as no

charges reach the ground through the current source (I_{Stim} = 0). In response to larger currents (Figure S15-B, right), the transmembrane potential V_m overcomes its threshold value and generates an action potential. Nevertheless, the potential across the OCEAN remains fully dictated by the stimulation current source and does not reflect the cell electrophysiological activity. When I_{Stim} is null, no current can flow from the cell to the OCEAN as $I_{Stim} = I_{PEDOT:PSS}$. Consequently, $V_{PEDOT:PSS}$, and therefore the optical signal of OCEANs, cannot be modulated by the cell potential in a pseudo-current clamp configuration.

In an ideal pseudo-voltage clamp configuration (Figure S15-C), the voltage source input impedance is null. As a result, current originating from the electrophysiological activity of a cell can flow through the PEDOT:PSS OCEAN, modulate the potential across its terminals, and, therefore, regulate its optical properties. In Figure S15-D, a bias voltage V_{Bias} of -0.1 V is applied between the ITO and the bath to simulate the operating voltage necessary to enhance the electro-optic sensitivity of the OCEAN. Interestingly, due to the large impedance of the PEDOT:PSS structure with respect to the rest of the circuit, most of V_{Bias} is transferred across the OCEAN and does not interfere with the transmembrane potential of the cell. Importantly, the cell transmembrane potential is not clamped by the external voltage source, compared to whole-cell voltage clamp experiments performed using the patch-clamp technique. In the event of an action potential, $V_{PEDOT:PSS}$ is directly modulated by the cell electrophysiological activity, demonstrating the feasibility of using OCEANs to perform electrophysiological studies. In consequence, OCEANs should be used in a pseudo-voltage clamp configuration to record the transmembrane potentials of cells without interfering with their physiology. Note that the seal resistance of a single OCEAN can theoretically be estimated by measuring its optical time constant in response to a voltage pulse, as the PEDOT:PSS capacitance loading kinetic is directly related to the quality of the cell-OCEAN interface.

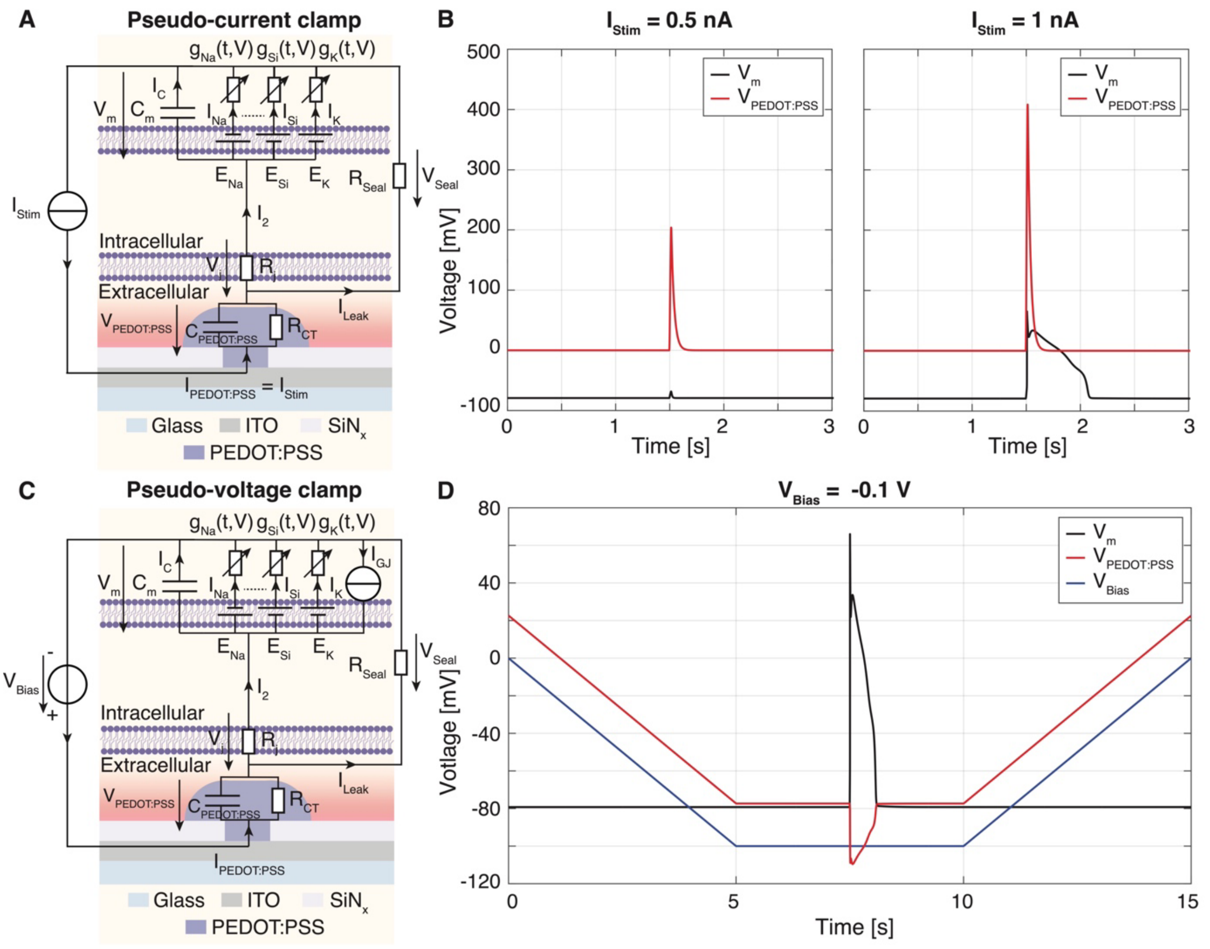

Figure S15: Modeling of cardiomyocyte action potential recording with OCEANs. A) Schematic representation of the cardiomyocyte-OCEAN interface according to a Luo Rudy model(*64*) in a pseudo current clamp configuration, where an ideal current source of infinite input impedance is connected between the OCEAN and the bath. B) Simulated voltage traces across the PEDOT:PSS OCEAN ($V_{PEDOT:PSS}$) and cell membrane (V_m) in response to a 20 ms long stimulation current pulse (I_{stim}) of 0.5 nA (left) and 1 nA (right) amplitude. 1 nA is enough to trigger the generation of an action potential in the ventricular cardiomyocyte, but this electrophysiological potential is not transferred across the PEDOT:PSS structure. C) Schematic illustration of the cardiomyocyte-OCEAN interface electrical equivalent circuit in a pseudo voltage clamp configuration, where an ideal voltage source is connected between the OCEAN and the bath to apply the optimal bias voltage. D) Computed voltage traces $V_{PEDOT:PSS}$ and V_m in response to the applied voltage bias V_{Bias} when a cardiomyocyte fires an action potential. In this case, the action potential is generated by an additional transmembrane current pulse (10 ms long, 0.4 nA amplitude), mimicking the contribution of the neighboring cells through gap junctions. For Figure S15-A and C, $C_{PEDOT:PSS}$ represents the capacitive charge transfer between the PEDOT:PSS and the bath, R_{CT} is the charge transfer resistance, R_j is the junctional resistance of the cell membrane, R_{Seal} is the seal resistance, and C_m is the non-junctional capacitance of the cell membrane. As defined in the Luo Rudy model,(*64*) fast sodium currents, slow inward currents, time-dependent potassium currents, time-independent potassium currents, plateau potassium currents, and background currents are represented by their respective transconductance $g_{Na}(t,V)$, $g_{Si}(t,V)$, $g_K(t,V)$, $g_{K1}(V)$, $g_{Kp}(V)$, and g_B and reversal potentials E_{Na}, E_{Si}, E_K, E_{K1}, and E_{Kp}. I_{GJ} represent the stimulation current originating from the neighboring cells through gap junctions and initiating the action potential. The numerical

values R_{Seal} = 200 MΩ and R_j = 500 MΩ were used in this model.(*44*) See the Supplementary Information, Section 5.3, for the numerical values of the remaining components of the circuit.

In summary, with MEAs, the amplifier's input impedance (*i.e.,* between the electrode and the bath/ground) must be much higher than the electrode impedance to minimize the voltage drop across the electrode-electrolyte interface. Ideally, it should be infinite so that the full amplitude of the electrophysiological signal originating from the cell and reaching the electrode would be transferred to the amplifier input without attenuation. With arrays of OCEANs, the sensing element is the OCEAN itself. Consequently, in an ideal scenario, the voltage drop across the PEDOT:PSS structure should not be minimized like with MEAs but maximized so that the entirety of the electrophysiological signal amplitude will contribute to modulating the scattering properties of the OCEAN. This condition is achieved by connecting the ITO to the ground or a voltage source (*i.e.,* null input impedance) when operating biases are needed.

The equations describing the pseudo-current and voltage clamp models presented in Figure S15 are introduced in the next two sub-sections. The last part discusses the assumptions made during the definition of the main components of the cell-OCEAN interfaces as well as the numerical values that were used in the models.

5.2 Equations defining the cardiomyocyte-OCEAN interface model

5.2.1 Pseudo current-clamp configuration

From the electrical equivalent circuit presented in Figure S15-A, the following equations can be defined:

$$V_{Seal} = V_m + V_j \quad \text{Equation S12}$$

Where V_{Seal} is the voltage drop across the seal resistance R_{Seal}, V_j across the junctional cell membrane, and V_m the transmembrane potential.

$$V_{Seal} = I_{Leak} \cdot R_{Seal} \quad \text{Equation S13}$$

Where I_{Leak} is the leaking current passing through R_{Seal}.

$$V_j = R_j \cdot I_2 = R_j \cdot (I_{Stim} - I_{Leak}) \quad \text{Equation S14}$$

Where R_j is the resistance of the junctional cell membrane, I_2 the current passing through the junctional cell membrane, and I_{Stim} the applied stimulation current.

From Equations S12 to S14, we can determine the following relationship:

$$I_{Leak} = \frac{(R_j \cdot I_{Stim} + V_m)}{R_{Seal} + R_j} \quad \text{Equation S15}$$

The current I_c passing through the cell membrane capacitance C_m can be expressed as:

$$I_c = I_{Stim} - (I_{Na} + I_{Si} + I_K + I_{K1} + I_{Kp} + I_B) - I_{Leak})$$ Equation S16

Where I_{Na}, I_{Si}, I_K, I_{K1}, I_{Kp}, and I_B area the fast sodium currents, slow inward currents, time-dependent potassium currents, time-independent potassium currents, plateau potassium currents, and background currents going through the cell membrane, as defined in the Luo Rudy model.(*64*)

As a result, the variation of transmembrane potential with time can be defined as:

$$\frac{dV_m}{dt} = \frac{I_c}{C_m}$$ Equation S17

Furthermore, because the current passing through the OCEAN-electrolyte interface $I_{PEDOT:PSS}$ is equal to the stimulation current I_{Stim}, the variation voltage across the OCEAN $V_{PEDOT:PSS}$ with time can be expressed as follows:

$$\frac{dV_{PEDOT:PSS}}{dt} = \frac{1}{C_{PEDOT:PSS}} \cdot \left(I_{Stim} - \frac{V_{PEDOT:PSS}}{R_{CT}}\right)$$ Equation S18

This system of differential equations was solved numerically using Matlab to find $V_{PEDOT:PSS}$ and V_m. The code implementing the pseudo-current clamp model is available in the dedicated data repository (see main Manuscript, Section 9).

5.2.2 Pseudo-voltage clamp configuration

The potential drop across the seal resistance V_{Seal} can be expressed as follows:

$$V_{Seal} = V_{Bias} - V_{PEDOT:PSS}$$ Equation S19

Where V_{Bias} is the applied voltage bias and $V_{PEDOT:PSS}$ the voltage drop across the OCEAN-electrolyte interface. The junctional potential V_j across the junctional cell membrane is therefore defined:

$$V_j = V_{Seal} - V_m$$ Equation S20

With V_m, the transmembrane potential of the cell of interest. Following Ohm's law, the resulting current I_2 passing through the junctional resistance of the cell membrane R_j is described as:

$$I_2 = \frac{V_j}{R_j}$$ Equation S21

And I_c, the current passing through the cell membrane capacitance as:

$$I_c = I_2 - (I_{Na} + I_{Si} + I_K + I_{K1} + I_{Kp} + I_B - I_{GJ})$$ Equation S22

Where I_{Na}, I_{Si}, I_K, I_{K1}, I_{Kp}, and I_B area the fast sodium currents, slow inward currents, time-dependent potassium currents, time-independent potassium currents, plateau potassium currents, and background currents going through the cell membrane, as defined in the Luo Rudy model.(*64*) I_{GJ} is the stimulating current coming from the neighboring cells through gap junctions and triggering the action potential.

The leaking current I_{Leak} passing through R_{seal} is defined as:

$$I_{Leak} = \frac{V_{Seal}}{R_{Seal}}$$ Equation S23

And the resulting current $I_{PEDOT:PSS}$ passing through the OCEAN-electrolyte interface as:

$$I_{PEDOT:PSS} = I_2 + I_{Leak}$$ Equation S24

The variation of V_m and $V_{PEDOT:PSS}$ in time followed the following relationships:

$$\frac{dV_m}{dt} = \frac{I_c}{C_m}$$ Equation S25

Where C_m is the cell membrane capacitance and:

$$\frac{dV_{PEDOT:PSS}}{dt} = \frac{1}{C_{PEDOT:PSS}} \cdot \left(I_{PEDOT:PSS} - \frac{V_{PEDOT:PSS}}{R_{CT}}\right)$$ Equation S26

Where R_{CT} the charge transfer resistance at the OCEAN-electrolyte interface

This system of differential equations was solved numerically using Matlab to find $V_{PEDOT:PSS}$ and V_m. The code implementing the pseudo-voltage clamp model is available in the dedicated data repository (see main Manuscript, Section 9).

5.3 Numerical values of the main components defining the cardiomyocyte-OCEAN interface model

The numerical values used for each component of the cell-OCEAN electrical equivalent circuit (Figure S15) are summarized in Table S4. A 1.4 µm in diameter OCEAN operating at V_{Bias} = -0.1 V was evaluated. Note that non-faradaic coupling at the PEDOT:PSS-electrolyte interface was assumed purely capacitive and modeled by $C_{PEDOT:PSS}$ for simplification purposes. Additionally, $R_{PEDOT:PSS}$ and R_S (Figure S6-A) were considered negligible and a charge transfer resistance R_{CT} was added to account for the faradaic charge transfer contributing to the discharging of $C_{PEDOT:PSS}$ at the OCEAN-electrolyte interface. Its value was experimentally measured to be 0.75 TΩ by cyclic voltammetry. In the pseudo-current clamp configuration, a value of 1 GΩ was used instead to keep discharge time constant in a reasonable window. Ion channel reversible potentials and transconductances were taken from the Luo Rudy model.(*64*)

Table S4: Numerical values used to model the cell-antenna interface. Table summarizing the numerical value of each component comprised in the cell-OCEAN interface electrical equivalent circuit

Component	Numerical value	Unit
$C_{PEDOT:PSS}$	26.6	pF
R_{CT}	0.75	TΩ
R_j	500	MΩ
R_{Seal}	200	MΩ
C_m	90.8	pF

5.4 Modeling the electro-optic characteristics of OCEAN with cells

Under total internal reflection illumination, an evanescent wave propagates vertically with an intensity that decays exponentially along the vertical axis. This wave is scattered by the antenna in an electrochemically dependent manner, enabling the wireless probing of local potential fluctuations. Due to the evanescent nature of the incident wave, only the portion of the cell in close proximity to the substrate is expected to interact with the light and potentially contribute to background scattering. For this reason, we focused on modeling the junctional cell membrane – 5 nm thick and located 20 nm above the antenna – and the cytoplasm (Figure S16-A). The spatial distribution of the electric field enhancement (Figure S16-B) demonstrates that the incident light contribution is minimal when it reaches the junctional membrane, validating our assumption. Figure S16-C to E further confirm that the electro-optic modulation of single OCEANs can be measured with minimal interference from the cell under total internal reflection. When an electrical stimulus from the cell modulates the scattering properties of the antenna (simulated as $V_{Stimulation}$ in Figure S16-C), the intensity of the scattered light is modulated, regardless of the presence of the cell on the antenna (Figure S16-D, red and blue curves). Although the absolute scattering cross-section is slightly reduced in the presence of the cell, the relative change in the optical signal remains nearly identical (Figure S16-E). Given the high brightness of the antenna, this small attenuation in absolute scattering cross-section is not expected to impact the electro-optic performance of the recordings. Overall, this model demonstrates the feasibility of using OCEANs in the presence of cells.

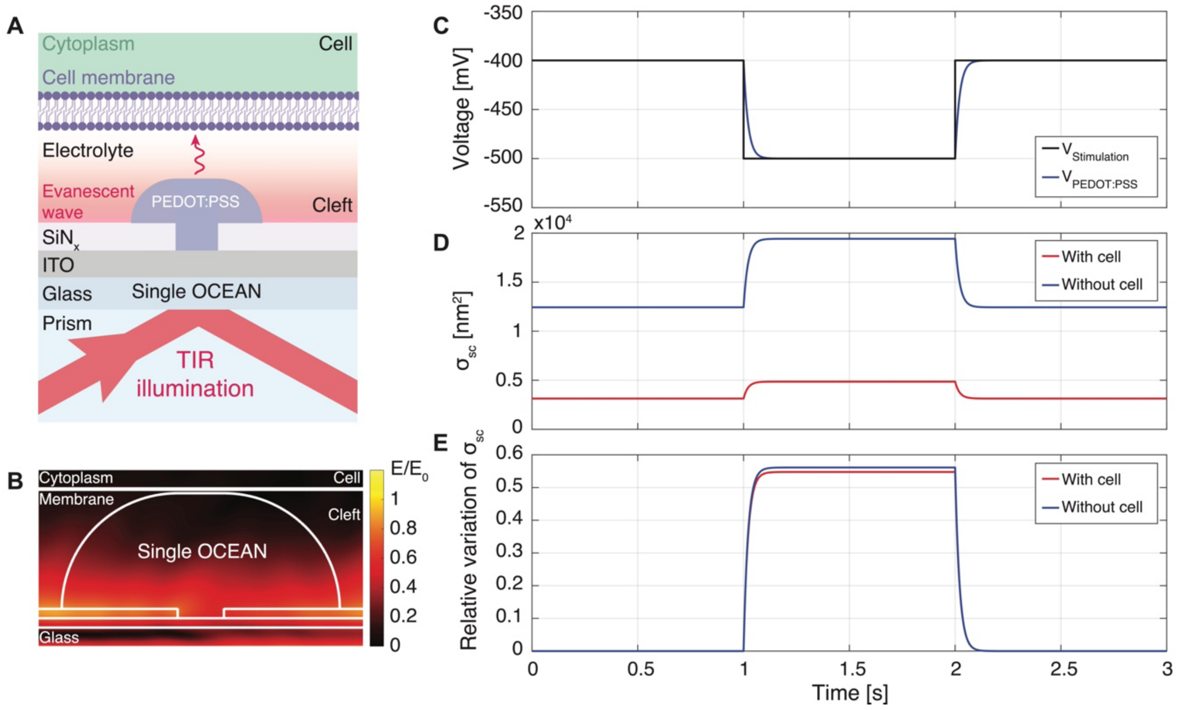

Figure S16: Modeling the electro-optic characteristics of a single OCEAN with cells. A) Schematic illustration of the simulated OCEAN-cell interface under total internal reflection illumination. B) Simulated spatial distribution of the electric field enhancement at the cell-antenna interface (E: Electric field, E_0: electric field of the incident illumination). C) Dynamic voltage anticipated across a 1500 nm in diameter OCEAN ($V_{PEDOT:PSS}$) in response to an applied -100 mV voltage pulse ($V_{Stimulation}$) with respect to a -0.4 V bias voltage. D) Absolute and E) relative dynamic electro-optic modulation of the system scattering cross-section (σ_{sc}) simulated in response to the stimulation voltage pulse described in C) with or without a cell covering the OCEAN. The relative variation of scattering cross section is defined as $\frac{\sigma_{sc}-\sigma_{sc,t=0}}{\sigma_{sc,t=0}}$, where $\sigma_{sc,t=0}$ is the scattering cross-section at the time t = 0 s.

Supplementary video S1: Electro-optic modulation of OCEANs during cyclic voltammetry. The optical signal scattered by OCEANs is modulated in response to voltage fluctuations in their surroundings during cyclic voltammetry in phosphate-buffered saline solution (t_{ED} = 45s).

REFERENCES AND NOTES

1. D. L. Bellin, H. Sakhtah, J. K. Rosenstein, P. M. Levine, J. Thimot, K. Emmett, L. E. P. Dietrich, K. L. Shepard, Integrated circuit-based electrochemical sensor for spatially resolved detection of redox-active metabolites in biofilms. *Nat. Commun.* **5**, 3256 (2014).

2. M. M. Mensack, J. B. Wydallis, N. S. Lynn, D. S. Dandy, C. S. Henry, Spatially resolved electrochemical sensing of chemical gradients. *Lab Chip* **13**, 208–211 (2013).

3. J. M. Rothberg, W. Hinz, T. M. Rearick, J. Schultz, W. Mileski, M. Davey, J. H. Leamon, K. Johnson, M. J. Milgrew, M. Edwards, J. Hoon, J. F. Simons, D. Marran, J. W. Myers, J. F. Davidson, A. Branting, J. R. Nobile, B. P. Puc, D. Light, T. A. Clark, M. Huber, J. T. Branciforte, I. B. Stoner, S. E. Cawley, M. Lyons, Y. Fu, N. Homer, M. Sedova, X. Miao, B. Reed, J. Sabina, E. Feierstein, M. Schorn, M. Alanjary, E. Dimalanta, D. Dressman, R. Kasinskas, T. Sokolsky, J. A. Fidanza, E. Namsaraev, K. J. McKernan, A. Williams, G. T. Roth, J. Bustillo, An integrated semiconductor device enabling non-optical genome sequencing. *Nature* **475**, 348–352 (2011).

4. R. Bounik, F. Cardes, H. Ulusan, M. M. Modena, A. Hierlemann, Impedance imaging of cells and tissues: Design and applications. *BME Front.* **2022**, 1–21 (2022).

5. J. Abbott, A. Mukherjee, W. Wu, T. Ye, H. S. Jung, K. M. Cheung, R. S. Gertner, M. Basan, D. Ham, H. Park, Multi-parametric functional imaging of cell cultures and tissues with a CMOS microelectrode array. *Lab Chip* **22**, 1286–1296 (2022).

6. J. Abbott, T. Ye, K. Krenek, R. S. Gertner, S. Ban, Y. Kim, L. Qin, W. Wu, H. Park, D. Ham, A nanoelectrode array for obtaining intracellular recordings from thousands of connected neurons. *Nat. Biomed. Eng.* **4**, 232–241 (2020).

7. J. Abbott, T. Ye, L. Qin, M. Jorgolli, R. S. Gertner, D. Ham, H. Park, CMOS nanoelectrode array for all-electrical intracellular electrophysiological imaging. *Nat. Nanotechnol.* **12**, 460–466 (2017).

8. J. Müller, M. Ballini, P. Livi, Y. Chen, M. Radivojevic, A. Shadmani, V. Viswam, I. L. Jones, M. Fiscella, R. Diggelmann, A. Stettler, U. Frey, D. J. Bakkum, A. Hierlemann, High-resolution CMOS MEA platform to study neurons at subcellular, cellular, and network levels. *Lab Chip* **15**, 2767–2780 (2015).

9. J. B. Kaushal, P. Raut, S. Kumar, Organic electronics in biosensing: A promising frontier for medical and environmental applications. *Biosensors* **13**, 1–48 (2023).

10. S. J. Wilks, S. M. Richardson-Burns, J. L. Hendricks, D. C. Martin, K. J. Otto, Poly(3,4-ethylenedioxythiophene) as a micro-neural interface material for electrostimulation. *Front. Neuroeng.* **2**, 591 (2009).

11. G.-T. Go, Y. Lee, D.-G. Seo, T.-W. Lee, Organic neuroelectronics: From neural interfaces to neuroprosthetics. *Adv. Mater.* **34**, e2201864 (2022).

12. M. J. I. Airaghi Leccardi, D. Ghezzi, Organic electronics for neuroprosthetics. *Healthc. Technol. Lett.* **7**, 52–57 (2020).

13. H. S. White, G. P. Kittlesen, M. S. Wrighton, Chemical derivatization of an array of three gold microelectrodes with polypyrrole: Fabrication of a molecule-based transistor. *J. Am. Chem. Soc.* **106**, 5375–5377 (1984).

14. Y. Jimbo, D. Sasaki, T. Ohya, S. Lee, W. Lee, F. Arab Hassani, T. Yokota, K. Matsuura, S. Umezu, T. Shimizu, T. Someya, An organic transistor matrix for multipoint intracellular action potential recording. *Proc. Natl. Acad. Sci. U.S.A.* **118**, 1–8 (2021).

15. D. Khodagholy, T. Doublet, P. Quilichini, M. Gurfinkel, P. Leleux, A. Ghestem, E. Ismailova, T. Hervé, S. Sanaur, C. Bernard, G. G. Malliaras, In vivo recordings of brain activity using organic transistors. *Nat. Commun.* **4**, 1575 (2013).

16. J. Rivnay, P. Leleux, M. Ferro, M. Sessolo, A. Williamson, D. A. Koutsouras, D. Khodagholy, M. Ramuz, X. Strakosas, R. M. Owens, C. Benar, J.-M. Badier, C. Bernard, G. G. Malliaras, High-performance transistors for bioelectronics through tuning of channel thickness. *Sci. Adv.* **1**, 1–5 (2015).

17. L. H. Jimison, S. A. Tria, D. Khodagholy, M. Gurfinkel, E. Lanzarini, A. Hama, G. G. Malliaras, R. M. Owens, Measurement of barrier tissue integrity with an organic electrochemical transistor. *Adv. Mater.* **24**, 5919–5923 (2012).

18. C. Yao, C. Xie, P. Lin, F. Yan, P. Huang, I.-M. Hsing, Organic electrochemical transistor array for recording transepithelial ion transport of human airway epithelial cells. *Adv. Mater.* **25**, 6575–6580 (2013).

19. G. C. Faria, D. T. Duong, A. Salleo, C. A. Polyzoidis, S. Logothetidis, J. Rivnay, R. Owens, G. G. Malliaras, Organic electrochemical transistors as impedance biosensors. *MRS Commun.* **4**, 189–194 (2014).

20. J. Rivnay, M. Ramuz, P. Leleux, A. Hama, M. Huerta, R. M. Owens, Organic electrochemical transistors for cell-based impedance sensing. *Appl. Phys. Lett.* **106**, 43301 (2015).

21. F. Bonafè, F. Decataldo, I. Zironi, D. Remondini, T. Cramer, B. Fraboni, AC amplification gain in organic electrochemical transistors for impedance-based single cell sensors. *Nat. Commun.* **13**, 1–9 (2022).

22. Z.-T. Zhu, J. T. Mabeck, C. Zhu, N. C. Cady, C. A. Batt, G. G. Malliaras, A simple poly(3,4-ethylene dioxythiophene)/poly(styrene sulfonic acid) transistor for glucose sensing at neutral pH. *Chem. Commun.* **4**, 1556–1557 (2004).

23. A.-M. Pappa, V. F. Curto, M. Braendlein, X. Strakosas, M. J. Donahue, M. Fiocchi, G. G. Malliaras, R. M. Owens, Organic transistor arrays integrated with finger-powered microfluidics for multianalyte saliva testing. *Adv. Healthc. Mater.* **5**, 2295–2302 (2016).

24. H. Liu, J. Song, Z. Zhao, S. Zhao, Z. Tian, F. Yan, Organic electrochemical transistors for biomarker detections. *Adv. Sci.* **11**, 2305347 (2024).

25. F. S. Alfonso, Y. Zhou, E. Liu, A. F. McGuire, Y. Yang, H. Kantarci, D. Li, E. Copenhaver, J. Bradley Zuchero, H. Müller, B. Cui, Label-free optical detection of bioelectric potentials using electrochromic thin films. *Proc. Natl. Acad. Sci. U.S.A.* **117**, 17260–17268 (2020).

26. Y. Zhou, E. Liu, Y. Yang, F. S. Alfonso, B. Ahmed, K. Nakasone, C. Forró, H. Müller, B. Cui, Dual-color optical recording of bioelectric potentials by polymer electrochromism. *J. Am. Chem. Soc.* **144**, 23505–23515 (2022).

27. C. Dingler, R. Walter, B. Gompf, S. Ludwigs, In situ monitoring of optical constants, conductivity, and swelling of PEDOT:PSS from doped to the fully neutral state. *Macromolecules* **55**, 1600–1608 (2022).

28. S. Doshi, D. Ludescher, J. Karst, M. Floess, J. Carlström, B. Li, N. Mintz Hemed, Y.-S. Duh, N. A. Melosh, M. Hentschel, M. Brongersma, H. Giessen, Direct electron beam patterning of electro-optically active PEDOT:PSS. *Nanophotonics* **13**, 2271–2280 (2024).

29. A. Habib, X. Zhu, U. I. Can, M. L. McLanahan, P. Zorlutuna, A. A. Yanik, Electro-plasmonic nanoantenna: A nonfluorescent optical probe for ultrasensitive label-free detection of electrophysiological signals. *Sci. Adv.* **5**, eaav9786 (2019).

30. J.-W. Jeon, P. A. Ledin, J. A. Geldmeier, J. F. Ponder Jr., M. A. Mahmoud, M. El-Sayed, J. R. Reynolds, V. V. Tsukruk, Electrically controlled plasmonic behavior of gold nanocube@polyaniline nanostructures: Transparent plasmonic aggregates. *Chem. Mater.* **28**, 2868–2881 (2016).

31. M. Locarno, D. Brinks, Analytical calculation of plasmonic resonances in metal nanoparticles: A simple guide. *Am. J. Phys.* **91**, 538–546 (2023).

32. J. Karst, M. Floess, M. Ubl, C. Dingler, C. Malacrida, T. Steinle, S. Ludwigs, M. Hentschel, H. Giessen, Electrically switchable metallic polymer nanoantennas. *Science* **374**, 612–616 (2021).

33. S. Chen, E. S. H. Kang, M. Shiran Chaharsoughi, V. Stanishev, P. Kühne, H. Sun, C. Wang, M. Fahlman, S. Fabiano, V. Darakchieva, M. P. Jonsson, Conductive polymer nanoantennas for dynamic organic plasmonics. *Nat. Nanotechnol.* **15**, 35–40 (2020).

34. A. Karki, Y. Yamashita, S. Chen, T. Kurosawa, J. Takeya, V. Stanishev, V. Darakchieva, S. Watanabe, M. P. Jonsson, Doped semiconducting polymer nanoantennas for tunable organic plasmonics. *Commun. Mater.* **3**, 1–8 (2022).

35. A. Karki, G. Cincotti, S. Chen, V. Stanishev, V. Darakchieva, C. Wang, M. Fahlman, M. P. Jonsson, Electrical tuning of plasmonic conducting polymer nanoantennas. *Adv. Mater.* **34**, e2107172 (2022).

36. L. Priest, J. S. Peters, P. Kukura, Scattering-based light microscopy: From metal nanoparticles to single proteins. *Chem. Rev.* **121**, 11937–11970 (2021).

37. C. F. Bohren, D. R. Huffman, *Absorption and Scattering of Light by Small Particles* (Wiley, 1983); https://onlinelibrary.wiley.com/doi/book/10.1002/9783527618156.

38. L. Rayleigh, On the transmission of light through an atmosphere containing small particles in suspension, and on the origin of the blue of the sky. *Lond. Edinb. Dublin Philos. Mag. J. Sci.* **47**, 375–384 (1899).

39. G. Mie, Beiträge zur Optik trüber Medien, speziell kolloidaler Metallösungen. *Ann. Phys.* **330**, 377–445 (1908).

40. J. Kolega, Phototoxicity and photoinactivation of blebbistatin in UV and visible light. *Biochem. Biophys. Res. Commun.* **320**, 1020–1025 (2004).

41. A. Peters, Z. Zhang, S. Faez, Dark-field light scattering microscope with focus stabilization. *HardwareX* **14**, e00424 (2023).

42. J. Rivnay, S. Inal, A. Salleo, R. M. Owens, M. Berggren, G. G. Malliaras, Organic electrochemical transistors. *Nat. Rev. Mater.* **3**, 1–14 (2018).

43. M. E. Spira, A. Hai, Multi-electrode array technologies for neuroscience and cardiology. *Nat. Nanotechnol.* **8**, 83–94 (2013).

44. J. Abbott, T. Ye, D. Ham, H. Park, Optimizing nanoelectrode arrays for scalable intracellular electrophysiology. *Acc. Chem. Res.* **51**, 600–608 (2018).

45. A. Hai, D. Kamber, G. Malkinson, H. Erez, N. Mazurski, J. Shappir, M. E. Spira, Changing gears from chemical adhesion of cells to flat substrata toward engulfment of micro-protrusions by active mechanisms. *J. Neural Eng.* **6**, 066009 (2009).

46. A. Fendyur, M. E. Spira, Toward on-chip, in-cell recordings from cultured cardiomyocytes by arrays of gold mushroom-shaped microelectrodes. *Front. Neuroeng.* **5**, 1–10 (2012).

47. N. Shmoel, N. Rabieh, S. M. Ojovan, H. Erez, E. Maydan, M. E. Spira, Multisite electrophysiological recordings by self-assembled loose-patch-like junctions between cultured hippocampal neurons and mushroom-shaped microelectrodes. *Sci. Rep.* **6**, 1–11 (2016).

48. I. H. Malitson, Interspecimen comparison of the refractive index of fused silica. *J. Opt. Soc. Am.* **55**, 1205 (1965).

49. T. A. F. König, P. A. Ledin, J. Kerszulis, M. A. Mahmoud, M. A. El-Sayed, J. R. Reynolds, V. V. Tsukruk, Electrically tunable plasmonic behavior of nanocube–polymer nanomaterials induced by a redox-active electrochromic polymer. *ACS Nano* **8**, 6182–6192 (2014).

50. L. Y. Beliaev, E. Shkondin, A. V. Lavrinenko, O. Takayama, Optical, structural and composition properties of silicon nitride films deposited by reactive radio-frequency sputtering, low pressure and plasma-enhanced chemical vapor deposition. *Thin Solid Films* **763**, 139568 (2022).

51. D. R. Lide, Concentrative properties of aqueous solutions: Density, refractive index, freezing point depression, and viscosity, in *CRC Handbook of Chemistry and Physics* (CRC Press, ed. 84, 2003); https://amazon.com/CRC-Handbook-Chemistry-Physics-84th/dp/0849304849.

52. R. A. Meyer, Light scattering from biological cells: Dependence of backscatter radiation on membrane thickness and refractive index. *Appl. Optics* **18**, 585–588 (1979).

53. Q. Zhang, L. Zhong, P. Tang, Y. Yuan, S. Liu, J. Tian, X. Lu, Quantitative refractive index distribution of single cell by combining phase-shifting interferometry and AFM imaging. *Sci. Rep.* **7**, 2532 (2017).

54. G. Evangelidis, E. Psarakis, Parametric image alignment using enhanced correlation coefficient maximization. *IEEE Trans. Pattern Anal. Mach. Intell.* **30**, 1858–1865 (2008).

55. C. M. Proctor, J. Rivnay, G. G. Malliaras, Understanding volumetric capacitance in conducting polymers. *J. Polym. Sci. Part B Polym. Phys.* **54**, 1433–1436 (2016).

56. J. L. Meyzonnette, J. Mangin, M. Cathelinaud, Refractive index of optical materials, in *Springer Handbook of Glass*, L. C. J. David Musgraves, Juejun Hu, Eds. (Springer, 2019), pp. 997–1045.

57. P. Drude, Zur Elektronentheorie der Metalle. *Ann. Phys.* **306**, 566–613 (1900).

58. H. A. Lorentz, *The Theory of Electrons and Its Applications to the Phenomena of Light and Radiant Heat* (B.G. Teubner and G.E. Stechert, 1916); https://archive.org/details/electronstheory00lorerich.

59. G. E. Jellison, F. A. Modine, Parameterization of the optical functions of amorphous materials in the interband region. *Appl. Phys. Lett.* **69**, 371–373 (1996).

60. W. Franks, I. Schenker, P. Schmutz, A. Hierlemann, Impedance characterization and modeling of electrodes for biomedical applications. *I.E.E.E. Trans. Biomed. Eng.* **52**, 1295–1302 (2005).

61. N. Kim, S. Kee, S. H. Lee, B. H. Lee, Y. H. Kahng, Y.-R. Jo, B.-J. Kim, K. Lee, Highly conductive PEDOT:PSS nanofibrils induced by solution-processed crystallization. *Adv. Mater.* **26**, 2268–2272 (2014).

62. B. X. E. Desbiolles, E. de Coulon, A. Bertsch, S. Rohr, P. Renaud, Intracellular recording of cardiomyocyte action potentials with nanopatterned volcano-shaped microelectrode arrays. *Nano Lett.* **19**, 6173–6181 (2019).

63. B. X. E. Desbiolles, E. de Coulon, N. Maïno, A. Bertsch, S. Rohr, P. Renaud, Nanovolcano microelectrode arrays: Toward long-term on-demand registration of transmembrane action potentials by controlled electroporation. *Microsyst. Nanoeng.* **6**, 1–12 (2020).

64. C. H. Luo, Y. Rudy, A model of the ventricular cardiac action potential. *Circ. Res.* **68**, 1501–1526 (1991).
